# Unveiling Comparative Genomic Trajectories of Selection and Key Candidate Genes in Egg-Type Russian White and Meat-Type White Cornish Chickens

**DOI:** 10.3390/biology10090876

**Published:** 2021-09-06

**Authors:** Alexandra S. Abdelmanova, Arsen V. Dotsev, Michael N. Romanov, Olga I. Stanishevskaya, Elena A. Gladyr, Andrey N. Rodionov, Anastasia N. Vetokh, Natalia A. Volkova, Elena S. Fedorova, Igor V. Gusev, Darren K. Griffin, Gottfried Brem, Natalia A. Zinovieva

**Affiliations:** 1 L.K. Ernst Federal Research Center for Animal Husbandry, 142132 Podolsk, Russia; preevetic@mail.ru (A.S.A.); asnd@mail.ru (A.V.D.); elenagladyr@mail.ru (E.A.G.); rodiand@ya.ru (A.N.R.); anastezuya@mail.ru (A.N.V.); natavolkova@inbox.ru (N.A.V.); igorgusev@mail.ru (I.V.G.); 2 School of Biosciences, University of Kent, Canterbury, Kent CT2 7NJ, UK; D.K.Griffin@kent.ac.uk; 3 K.I. Skryabin Moscow State Academy of Veterinary Medicine and Biotechnology, 23 Akademika Skryabina St., 109472 Moscow, Russia; 4 Russian Research Institute of Farm Animal Genetics and Breeding—Branch of the L.K. Ernst Federal Research Center for Animal Husbandry, Pushkin, 196601 St. Petersburg, Russia; olgastan@list.ru (O.I.S.); osot2005@yandex.ru (E.S.F.); 5 Institute of Animal Breeding and Genetics, University of Veterinary Medicine, 1210 Vienna, Austria; gottfried.brem@vetmeduni.ac.at

**Keywords:** selection signatures, genomic regions, candidate genes, gene ontology, gene richness, chicken, SNPs, Russian White breed, White Cornish breed

## Abstract

**Simple Summary:**

The search for genomic regions of putative selective signaling is instrumental in obtaining information about selection history in various species and populations. Domestic animals are subject to long-term artificial selection that leaves certain footprints in their genomes one can explore using genome-wide SNP screen. We examined here genomes of two contrasting chicken breeds, the native egg-type Russian White and meat-type White Cornish. Using three statistics, we identified genomic regions under putative selection, both breed-specific and shared between two breeds, that harbor key candidate genes for economically important traits. Our findings will be useful in further understanding selection history and genomic diversity in domestic chickens that would be pivotal in their productive breeding.

**Abstract:**

Comparison of genomic footprints in chicken breeds with different selection history is a powerful tool in elucidating genomic regions that have been targeted by recent and more ancient selection. In the present work, we aimed at examining and comparing the trajectories of artificial selection in the genomes of the native egg-type Russian White (RW) and meat-type White Cornish (WC) breeds. Combining three different statistics (top 0.1% SNP by *F*_ST_ value at pairwise breed comparison, hapFLK analysis, and identification of ROH island shared by more than 50% of individuals), we detected 45 genomic regions under putative selection including 11 selective sweep regions, which were detected by at least two different methods. Four of such regions were breed-specific for each of RW breed (on GGA1, GGA5, GGA8, and GGA9) and WC breed (on GGA1, GGA5, GGA8, and GGA28), while three remaining regions on GGA2 (two sweeps) and GGA3 were common for both breeds. Most of identified genomic regions overlapped with known QTLs and/or candidate genes including those for body temperatures, egg productivity, and feed intake in RW chickens and those for growth, meat and carcass traits, and feed efficiency in WC chickens. These findings were concordant with the breed origin and history of their artificial selection. We determined a set of 188 prioritized candidate genes retrieved from the 11 overlapped regions of putative selection and reviewed their functions relative to phenotypic traits of interest in the two breeds. One of the RW-specific sweep regions harbored the known domestication gene, *TSHR*. Gene ontology and functional annotation analysis provided additional insight into a functional coherence of genes in the sweep regions. We also showed a greater candidate gene richness on microchromosomes relative to macrochromosomes in these genomic areas. Our results on the selection history of RW and WC chickens and their key candidate genes under selection serve as a profound information for further conservation of their genomic diversity and efficient breeding.

## 1. Introduction

Since the middle of the last century, the poultry industry has focused on the exploitation of a few highly specialized, productive lines selected for egg or meat (broiler) production traits. In the chicken *(Gallus gallus*; GGA), this has led to a drastic decline in the number and population size of native breeds that were extensively used for agricultural production in the past. Many native breeds are now at the risk of being lost, while potentially harboring significant historical footprints of selection for economically important phenotypes in their genomes. Conservation and molecular genetic characterization of biodiversity among domestic animal and poultry species used for food production are two of the key objectives to ensure the sustainability of local and regional agricultural production systems [1,2,3,4,5,6,7]. Genomic tools and resources for poultry-related research have advanced and been immensely enriched since the time of the first chicken reference genome sequence publication [8].

Two main evolutionary lineages of domestic chickens are represented by egg-type and meat-type breeds [9]. One of the distinctive native egg-type chicken breeds is the Russian White (RW), developed for egg production in the former USSR in 1929–1953 by crossing local low-productive hens with White Leghorn roosters of Danish, British, and American origins [10]. Before 1965, the RW was a major chicken breed employed for egg production in the USSR. In 1975, the number of RW chickens was 29.73 million heads; however, there was a dramatic drop by 1980 to 4.4 million [10], with a further essential population reduction in later years.

Historically, RW chickens were brought to the Russian Research Institute of Farm Animal Genetics and Breeding (RRIFAGB), Pushkin, from the Leningradskaya egg production plant in 1952. Along with the selective breeding for high egg performance in 1952–1999, the RW chickens were selected for tolerance to low temperatures. This gave rise to establishing in 1957 and developing an inbred RS subpopulation selected for cold tolerance of chicks [11,12]. The breeding RS stock was kept at 15–22 °C during the first five days after hatch, with a gradual temperature lowering to 14 and 11 °C by 21–30 days. Adult individuals were kept in winter at a temperature below 0 °C, while safeguarding the laying performance at a high level [13]. As a result of the long-term selection for critically low temperatures, the RS phenotypes were characterized by exceptional cold tolerance of neonatal chicks; their snowy-white down at day-old presumably caused by a recessive mutant gene, *sw*, for snow-white down [14,15]; and elevated resistance to a few neoplastic diseases, such as Marek’s disease, leukemia, and carcinomas [11]. The current RG population was more recently derived from the RS as a result of a one-time telic crossing with White Leghorns in 2006 not selected further for cold tolerance and subjected to random mating in 2000–2013 [12,16,17,18].

Presently, the small RG population of cold-tolerant RW chickens (25 males and 234 females) is kept in the Genetic Collection of Rare and Endangered Chicken Breeds of the RRIFAGB. In the last two decades, the RW breeding program has been aimed at improved egg production and egg weight and increased yield of vaccine raw material, i.e., extraembryonic (allanto-amniotic) fluid and titers of vaccine viruses in it while maintaining cold tolerance of neonatal chickens [15,19,20]. There is also one more RW population maintained at the All-Russian Poultry Research and Technological Institute (ARPRTI) collection farm, Sergiev Posad.

Because of importance of the yield of extraembryonic fluid (YEF) to produce vaccines, in-depth molecular genetic studies were undertaken to pinpoint markers for YEF in the RW population at RRIFAGB. Its examination for the effects of indels in the *PRL* (prolactin) and *DRD2* (dopamine receptor D2) genes on YEF showed significant associations of the PRL insertion variant with greater YEF and egg weight (EW) [16]. In a genome-wide association study (GWAS) using the Illumina Chicken 60K SNP iSelect BeadChip, several suggestive SNP loci and candidate genes were detected for YEF as well as EW, egg number, age at first egg, body weight, and day-old chick down color [15]. The RW demographic history was explored at the genomic level using the same Illumina BeadChip. Within the current RG population, a heterogeneity of SNP genotypes was revealed, suggesting its subdivision into four subgroups: two relatively homogenous, one heterogenous, and one phylogenetically closer to the historical RS subpopulation. The latter, in turn, was distinguished by more numerous monomorphic markers and longer linkage disequilibrium (LD) regions as well a greater number of runs of homozygosity (ROHs), their greater mean length, and a higher ROH-based inbreeding coefficient. The RG population had the smallest LD values and the largest effective population size [12,17,18]. In an additional GWAS using the Illumina Chicken 60K SNP iSelect BeadChip, nine SNPs were reported in the RG population that demonstrated significant associations with egg production traits and coincided with ROHs [21]. A sampling of RW chickens was also tested for copy number variation (duplication) at the *AvBD7* (avian beta-defensin 7) gene relevant to the innate immune system, with a ratio of three duplication carriers to four nonduplication individuals [22].

An old meat-type breed of White Cornish (WC) chickens was created by crossing the English Game breed with Aseel or Malay fowls. It was recognized as a breed in England in 1886 under the name of Indian Game. Unlike RW chickens, the WC breed has been specifically selected for growth performance, feed efficiency, and carcass traits [23]. Such intensive selection for the limited number of traits in meat-type (broiler) chickens has led to the occurrence of increased frequencies of undesirable traits such as low fertility, reduced fitness, increased disease susceptibility, skeletal weakness, etc. [23,24,25,26]. In Russia, a few WC crossbred strains are maintained in the RRIFAGB gene pool collection and have been explored in the genome-wide scanning studies using the Illumina BeadChip [17,18]. They demonstrated a reduced genetic diversity because of small population size, and lower ROH metrics due to crossbreeding effects [17]. A small population of purebred WC chickens is also kept in the ARPRTI gene pool collection.

Comparison of genomic footprints in two chicken breeds with different selection history, i.e., native egg-type RW and meat-type WC, may be helpful in elucidating genomic regions that have been targeted by recent and more ancient selection. Detecting regions of the genome that have undergone artificial selection will expand our understanding of the domestication history and selective breeding in chickens. Identification of genes within such genomic regions will aid in development of effective breeding programs based on marker-assisted and genomic selection.

Different methodologies have been utilized for defining regions of the genome that exhibit evidence of having been under selection. One of the most used methods is based on estimating the fixation index (*F*_ST_), which quantifies the differences in allele frequencies between populations [27,28]. The fixation index is a single SNP test, which is routinely used for identifying highly differentiated alleles [29]. Genetic differentiation between populations is expected to be low in neutral regions of the genome or in regions under balancing selection, whereas high genetic differentiation indicates regions that have undergone divergent selection among populations. Another metric used for determining genomic regions that have been subjected to selection is the hapFLK analysis. This is a haplotype-based statistic for detecting positive selection using multiple population data [30,31]. For the calculation of hapFLK, both population structure and haplotype information are taken into consideration. The identification of ROH islands was proposed as one more useful indicator of selection signals in livestock populations [32,33]. ROH islands are genomic regions with high homozygosity around a selected locus that might harbor targets of positive selection [34]. Combining different statistics for defining selection signatures can improve the reliability of identified genomic regions [35,36].

Several genome-wide studies have been undertaken to detect the genomic footprints of artificial selection in large numbers of chicken breeds (e.g., [37,38,39,40,41]). Despite the initial chicken genome research efforts undertaken so far at the RRIFAGB [17,18], a comparative in-depth search for selection sweeps in chicken breeds of Russian origin at the genome-wide level have not been carried out and would be much anticipated.

In the present study, we aimed to examine and compare the trajectories of artificial selection in the genomes of two chicken breeds with different selection history and breeding objectives, i.e., the native egg-type RW and meat-type WC breeds. We performed the structural annotation of discovered genomic regions, suggesting multiple key candidate genes associated with egg/meat production and other economically important traits. Our results elucidate the breeding history of RW and WC chickens and provide a useful information for further conservation of their genomic diversity and sustainable breeding.

## 2. Materials and Methods

### 2.1. Experimental Birds and Ethics Statement

**Figure 1:**
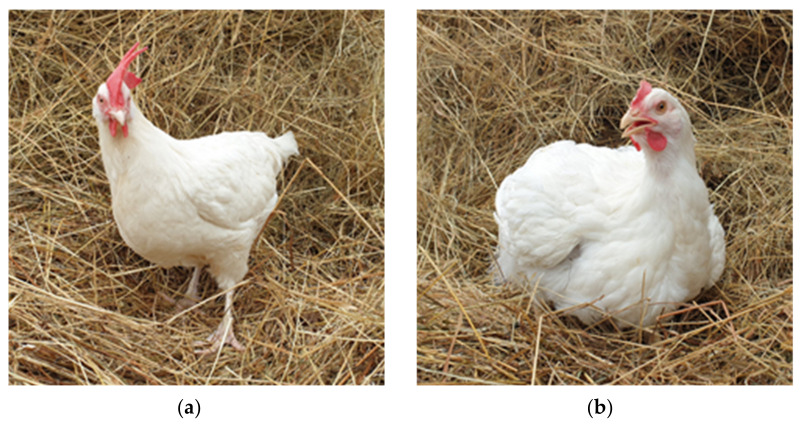
Two studied chicken breeds: Russian White (**a**) and White Cornish (**b**).

Young chickens of the RW and CW breeds (Figure 1) were purchased and housed in the Gene Pool Collection of Farm and Wild Animals and Birds of the L.K. Ernst Federal Research Center for Animal Husbandry (LKEFRCAH) with the aim of establishing an F_2_ resource population to be used for subsequent GWAS experiments. RW chickens of the cold-tolerant line were provided by the RRIFAGB. Nonpedigreed crossbred CW chickens were provided by the Breeding Genetic Center “Smena”, a subsidiary of the Federal Science Center for Poultry Science, of the Russian Academy of Sciences. Individuals of both breeds were grown to the age of 6 months and used to produce F_1_ generation. No chickens used in the present studies were subject to further pure breeding. Feather samples were collected by trained lab personnel following the LKEFRCAH ethical guidelines for minimizing any possible bird discomfort or distress.

### 2.2. Sampling and DNA Extraction

Feather samples were obtained from 54 individuals including 31 samples of the RW chickens and 23 samples of the WC breed. DNA extraction was performed using Nexttec columns (Nexttec Biotechnologie GmbH, Leverkusen, Germany) according to manufacturer’s instructions. Concentration of dsDNA solutions was determined using a Qubit 3.0 fluorometer (Thermo Fisher Scientific, Wilmington, DE, USA). To check the purity of extracted DNA, OD260/280 ratio was tested using NanoDrop-2000 (Thermo Fisher Scientific, Wilmington, DE, USA).

### 2.3. SNP Genotyping and Quality Control

Individual sample genotyping was carried out using Chicken 50K_CobbCons chip (Illumina, San Diego, CA, USA). Input files were created using R software [42]. To determine valid genotypes for each SNP, we set a cut-off for the GenCall (GC) and GenTrain (GT) scores of 0.5 [43]. Using PLINK 1.9 software [44], SNP quality control was performed. All chicken genotypes passed the filtering for genotyping efficiency (--mind 0.2). Only SNPs located on autosomes from GGA1 to GGA28 were used for analysis, and SNPs genotyped in less than 90% of the samples (--geno 0.1) were excluded from the analysis. The final data set used for analyzing signatures of selection included 44,728 autosomal SNPs. Additional filters for LD values were used for performing calculation of genetic diversity, principal component analysis (PCA), Neighbor-Net tree construction, and admixture clustering that resulted in 25,768 SNPs. One SNP from each pair of neighbored SNPs where LD (*r*^2^) value exceeded 0.5 within 50 SNP windows was removed using--indep-pairwise 50 5 0.5 flag, where 50 is size of the sliding window, 5 is the number of SNPs shifted in each step, and 0.5 is the *r*^2^ threshold. Positions of SNPs were assigned according to the GGA reference genome assembly GRCg6a [45].

### 2.4. Genetic Diversity

PLINK 1.9 [44] was used to evaluated within-population genetic diversity. We calculated the observed heterozygosity (*H_O_*), unbiased expected heterozygosity (*_U_H_E_*) [46], rarefied allelic richness (*A_R_*) [47], and inbreeding coefficient (*_U_F*_IS_) based on the unbiased expected heterozygosity by using R package diveRsity [48].

### 2.5. PCA, Neighbor-Net, and Admixture

Procedures of PCA, Neighbor-Net clustering, and model-based clustering (Admixture) were exploited to select the individuals for the analysis of selection sweeps. PCA was performed using PLINK v1.9 software. An R package ggplot2 was used to visualize the results [49]. Pairwise identical-by-state (IBS) distances calculated in SplitsTree4 [50] were used to construct a Neighbor-Net tree. Admixture v1.3 software [51] was employed for genetic admixture analysis and an R package pophelper [52] for plotting the results. A standard admixture cross-validation procedure [53] was used to calculate the number of ancestral populations (K). The assumed number of K corresponded to the lowest value of cross-validation (CV) error as compared to other K values.

### 2.6. Selection Signature Analysis

Three different statistics were used for detecting the signatures of selection in the genome of chicken: calculation of *F*_ST_ values for each SNP when comparing pairs of breeds, hapFLK analysis, and estimation of the ROH islands, which were overlapped among different animals within each breed.

#### 2.6.1. F_ST_ Analysis

Pairwise *F*_ST_-based genetic differentiation [53] was estimated between all SNPs using PLINK 1.9. We applied a low threshold for minor allele frequency (MAF) of less than 5% (--maf 0.05), because the filtering of SNPs based on MAF may affect the probability of identifying alleles related to selection [54]. The dataset used for *F*_ST_ analysis included 44,728 autosomal SNPs. The top 0.1% *F*_ST_ values were used to represent a selection signature, as was previously proposed by Kijas et al. [55] and Zhao et al. [56].

#### 2.6.2. Runs of Homozygosity

A window-free method for consecutive SNP-based detection, i.e., consecutive runs method [57] implemented in an R package detectRUNS [58], was used for the estimation of ROHs. We allowed one SNP with missing genotype and up to one possible heterozygous genotype in one run to avoid the underestimation of the number of ROHs that were longer than 8 Mb [59]. Because of strong linkage disequilibrium (LD), typically extending up to about 100 kb [60], and for excluding short and very common ROHs, we set the minimum length for an ROH at 500 kb. For minimizing false-positive results, we calculated the minimum number of SNPs (*l*) as was initially evaluated by Lencz et al. [61] and later modified by Purfield et al. [62]:

$$l=\frac{log_{e}\frac{\alpha }{n_{s}\cdot n_{i}}}{log_{e}\left(1-\bar{het}\right)},$$

where *n_s_* is number of genotyped SNPs per individual, *n_i_* is number of genotyped individuals, *α* is percentage of false-positive ROHs (set to 0.05 in our study), and $\bar{het}$ is mean heterozygosity across all SNPs. In our case, the minimum number of SNPs was 23.

We estimated the number and length of ROHs for each individual and then averaged these per individual within each breed. Additionally, we computed the genomic inbreeding coefficient based on ROH (*F*_ROH_) as the ratio of the sum of the length of all ROHs per animal to the total autosomal SNP coverage (0.94 Gb).

Number of ROHs in the genomes of two studied breeds was examined using the following ROH length classes: 0.5–1, 1–2, 2–4, 4–8, 8–16, and >16 Mb. To define the proportion of genome covered by different ROH segments, we calculated the sum of ROHs for the following different minimum lengths: >0.5, >1, >2, >4, >8, and >16 Mb.

Putative ROH islands were defined as overlapping homozygous regions that shared by more than 50% of analyzed individuals within each breed, as this was suggested in other studies [63,64]. We applied the threshold of 0.3 Mb for the minimal overlapping length size, because it was previously shown that shorter segments of 0.3–1 Mb are predominant in genome of white layers [41].

#### 2.6.3. HapFLK Analysis

To detect the signatures of selection through haplotype differentiation among the studied breeds, we also employed hapFLK 1.4 program [30,31]. The number of haplotype clusters per chromosome was determined in fastPHASE by using cross-validation-based estimation and was set at 35 [65]. For detailed analyses, we selected the hapFLK regions containing at least one SNP with *p*-value threshold of 0.01 (−log10(*p*) > 2).

### 2.7. Search for Genes and QTLs Localized within Identified Genomic Regions

For candidate gene mining in the genomic regions under putative selection, we used the genomic localization of the regions as detected by three different statistics, i.e., *F*_ST_, ROH, and hapFLK methods. We prioritized those regions that were overlapped and revealed at least by two different techniques. Borders of these regions as localized in the GRCg6a reference assembly chromosomes were used as a query list for retrieving chicken genes and their human orthologs using the web-based Ensembl Genes release 103 database and Ensembl BioMart data mining tool [66]. Results retrieved from the Ensembl BioMart browser for each genomic region of selection signatures were manually sieved and compared to relevant published investigations to identify main candidate genes and other genes of interest.

Additionally, a broader gene mining was carried out that also included the regions found by one method to expand the candidate list with more previously discovered and important genes. Where needed for comparisons with other studies, we used the UCSC liftOver tool [67] to convert genome coordinates between earlier chicken genome builds and the reference assembly GRCg6a. If an older build could not be directly compared to GRCg6a, a sequential lift of coordinates between assemblies was applied.

For specifying the presence of quantitative trait loci (QTLs) and associated genes overlapped with the identified genomic regions, we also analyzed a publicly available chicken database, QTLdb [68].

### 2.8. Gene Ontology Mining

To perform functional annotation and gene ontology (GO) term enrichment analysis within the determined selective sweep regions, we exploited the Database for Annotation, Visualization, and Integrated Discovery (DAVID) v6.8 [69,70]. Using our in-house GO mining pipeline consisted of the BioMart and DAVID tools, the gene and background lists were generated following the procedure described in detail elsewhere [71]. Briefly, chicken–human orthologs of the maximum orthology confidence served as entries for the gene and background lists, applying the 70% gene identity threshold to the gene list and reducing the background list to chromosomes containing the selection footprints. Significant annotation clusters were selected using an enrichment score of more than 1.3 and a *p*-value < 0.05.

## 3. Results

### 3.1. Genetic Diversity

The RW chickens were characterized by significantly lower level of genetic diversity assessed by the level of unbiased expected heterozygosity (*_U_H_E_* = 0.339 vs. 0.383, *p* < 0.001) and allelic richness (*A_R_* = 1.937 vs. 1.982, *p* < 0.001) as compared to the WC breed. We observed significant deviation in the number of heterozygotes from the Hardy–Weinberg equilibrium in both studied populations. The negative value of the inbreeding coefficient *_U_F*_IS_ indicates a slight excess of heterozygotes in the RW population (*_U_F*_IS_ = –0.016), while the WC breed showed a slighter deficiency of heterozygotes (*_U_F*_IS_ = 0.009) than it would be expected under Hardy–Weinberg equilibrium (Table 1).

### 3.2. Breed Relationship and Admixture

**Figure 2:**
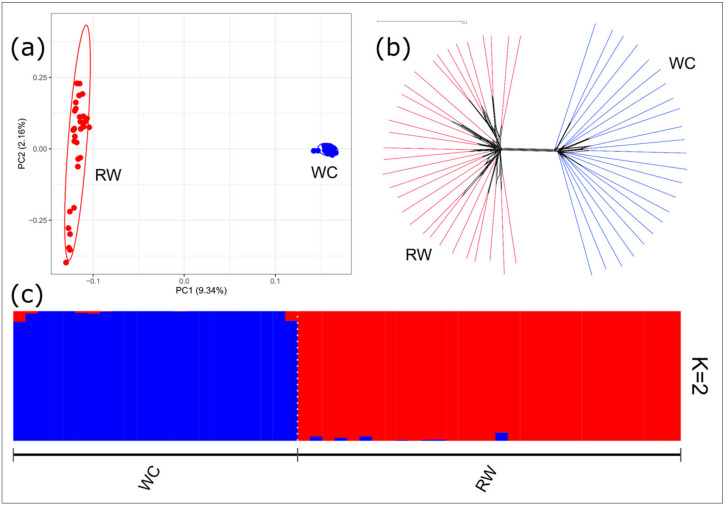
Genetic relationships between the Russian White (RW) and White Cornish (WC) chicken populations: (**a**) a principal component analysis (PCA) plot showing the distribution of RW and WC individuals in the dimensions of two coordinates, i.e., the first (PC1; *X*-axis) and second (PC2; *Y*-axis) principal components, with percentage of total genetic variability, which can be explained by each of the two components, being indicated within the parentheses; (**b**) a Neighbor-Net tree constructed based on the *F*_ST_ genetic distances among the studied populations; and (**c**) an admixture plot representing cluster structure of the studied populations if the number of clusters K = 2.

As assessed by PCA, the first component, which is responsible for 9.34% of the genetic variability, clearly differentiated the RW chickens from the WC breed (Figure 2a). The neighbor-joining tree constructed based on pairwise IBS distances showed the breed-specific distribution of individuals between the two groups (Figure 2b). The calculations of CV error for the different number of clusters (from 1 to 5) showed that the probable number of clusters (K) was equal to 2 (Figure S1). The admixture clustering at K = 2 clearly distinguished the RW and WC populations at K = 2 (Figure 2c), indicating a very low level of admixture among the studied populations.

### 3.3. ROH Distribution in the Genomes of Studied Chicken Breeds

We revealed a lower mean number of ROH segments in the RW breed genome as compared to the WC breed (109.10 vs. 119.09). In contrast, RW chickens had a greater coverage of genome by ROHs (183.41 vs. 161.97 Mb), which resulted in a higher value of inbreeding coefficient calculated based on ROHs (*F*_ROH_ = 0.195 vs. 0.172) (Table 2).

**Figure 3:**
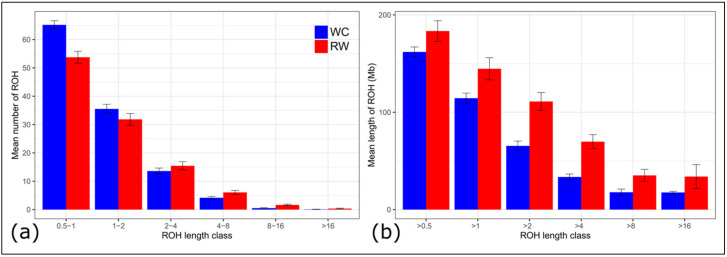
Descriptive statistics of the runs of homozygosity (ROH) by ROH length class in the breeds of Russian White (RW) and White Cornish (WC) chickens: (**a**) mean number of ROHs (Y-axis) by ROH length class (X-axis; 0.5–1, 1–2, 2–4, 4–8, 8–16, and >16 Mb); (**b**) overall mean length of ROHs (Y-axis) by ROH length class (X-axis; >0.5 Mb, >1 Mb, >2 Mb, >4 Mb, >8 Mb, and >16 Mb).

Short ROH segments (0,5–1 Mb) were the most distributed throughout the genome and accounted for 49.26% and 54.73% of all ROHs identified in the WC and RW breeds, respectively. The proportion of ROH segments of the greater length (4–8, 8–16, and >16 Mb), typically caused by inbreeding to a more recent ancestor, was higher in RW as compared to WC (5.56 vs. 3.50% for 4–8 Mb; 1.51 vs. 0.44 for 8–16 Mb; and 0.33 vs. 0.07 for ROH > 16 Mb). The genome coverage by the longer ROH segments was greater in RW than that in WC (18.9 vs. 9.69%; 0.66 vs. 0.13%; and 18.49 vs. 10.87%, respectively) (Figure 3, Table S1).

### 3.4. Analysis of the Signatures of Selection

To detect the signatures of selection in the genome of the studied chicken populations, we calculated *F*_ST_ values for each SNP, performed hapFLK analysis, and estimated ROH islands, which were overlapped among different individuals within each population.

**Figure 4:**
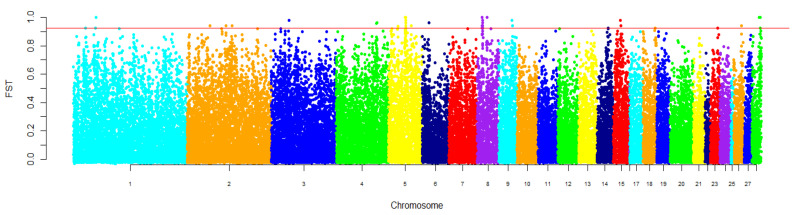
Genomic distribution of *F*_ST_ values estimated between the Russian White and White Cornish populations. Values for the *X*-axis are chicken autosomes (breadth of autosomes corresponds to their length) and those for the *Y*-axis are *F*_ST_ values. SNPs were plotted relative to their positions within each autosome. The threshold, which was estimated as the top 0.1% for *F*_ST_ values, is indicated by a horizontal line.

Average pairwise *F*_ST_ value between the studied populations equaled 0.152. We identified 45 SNPs with *F*_ST_ beyond the cut-off value (top 0.1%, *F*_ST_ > 0.92) that were distributed among twelve autosomes (GGA1, GGA2, GGA3, GGA4, GGA5, GGA6, GGA8, GGA9, GGA15, GGA18, GGA26, and GGA28). The greatest number of SNPs was found on GGA5 (16 SNPs) and GGA8 (10 SNPs). Fourteen SNPs localized on GGA5 and all of SNPs on GGA8 were presented by blocks of neighboring SNPs (Figure 4, Table S2).

We found the overlapping ROH islands observed in more than 50% of samples within each population (Table S3, Figure S2). We detected 23 ROH islands in the WC breed, which covered 25.987 Mb of genome and were distributed among nine chromosomes (GGA1, GGA2, GGA3, GGA4, GGA5, GGA7, GGA8, GGA18, and GGA28). Average length of ROH islands was 1.130 Mb, with their size ranging from 0.405 to 3.240 Mb. Eighteen ROH islands covering 14.530 Mb of the genome and distributed among seven chromosomes (GGA1, GGA2, GGA3, GGA4, GGA5, GGA8, and GGA9) were found in the RW population. Length of ROH islands varied between 0.301 and 2.472 Mb and averaged 0.807 Mb. Most of the identified ROH islands were population-specific, except for two ROH islands on GGA1 and GGA2, which partly overlapped in the two studied populations. The ROH islands on GGA1 covered a region between 143,268,071 and 144,065,046 in the genome of the WC breed and between 143,047,114 and 143,929,272 in the genome of the RW breed. The positions of ROH islands on GGA2 were 78,935,731 to 79,963,158 for the WC breed and 79,022,396 to 79,487,897 for the RW chickens.

**Figure 5:**
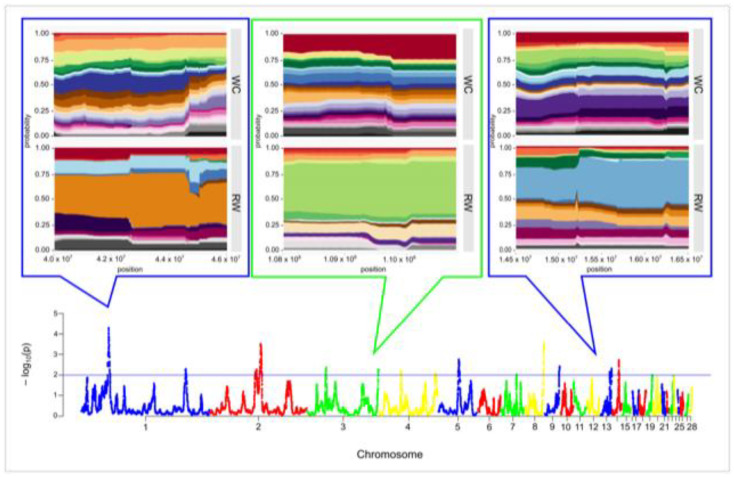
Signatures of selection in the genomes of the studied Russian White (RW) and White Cornish (WC) chicken populations based on the hapFLK statistics. Values for the *X*-axis are chicken autosomes, and those for the *Y*-axis are values of statistical significance (−log_10_ *p*-values). The blue line indicates threshold of significance at *p* < 0.01 (i.e., −log_10_(*p*) > 2). Magnified plots of the few most representative chromosome areas containing the hapFLK regions are presented above the plot. Values for the *X*-axis are the genomic positions on corresponding autosome. Each color band corresponds to one haplotype cluster, and the height of a band shows the cluster frequency. Magnified plots for all 15 putative regions identified by hapFLK analysis are presented in Figure S3.

The hapFLK analysis resulted in identification of 15 putative regions affected by selection, which were distributed among 10 autosomes and covered 20.095 Mb of the chicken genome. Length of the putative regions under selection pressure ranged between 0.022 and 3.764 Mb and averaged 1.340. Twelve regions were breed-specific, including four regions on GGA1, GGA7, GGA13, and GGA14 in the WC breed (coverage of 2.412 Mb) and eight regions on GGA1, GGA3, GGA4, GGA5, GGA8, GGA9, and GGA13 (coverage of 10.572 Mb) in RW chickens. Three genomic regions identified by hapFLK analysis on GGA2 (positions from 69,808,276 to 72,600,534 and from 76,552,199 to 80,316,420) and GGA3 (positions from 29,203,776 to 29,759,199) were common for both populations (Figure 5 and Figure S3, Table S4). Three genomic regions with the significance level *p* < 0.001, which were detected on GGA1 (positions from 41,095,175 to 44,484,517) and GGA8 (positions from 28,777,070 to 30,174,896) in RW chickens, as well as one common region on GGA2 (positions from 76,552,199 to 80,316,420) were defined as strongest hapFLK regions.

Comparing genomic localization of the regions under putative selection detected by three different statistics (*F*_ST_, ROHs, and hapFLK) revealed the presence of 11 overlapped regions, which were identified at least by two different methods (Table 3). The identification of these regions by at least two detection methods implies that such regions are likely to bear true signatures of selection.

Thirty-one genomic regions identified in our study by either one or more metrics were overlapped with regions, which were previously detected in other investigations in different chicken breeds (e.g., [15,38,39,40,41,72,73,74,75]). These selection sweeps were localized on GGA1 (three regions), GGA2 (seven regions), GG3 (three regions), GGA4 (five regions), GGA5 (three regions), GGA7 (two regions), GGA13 (two regions), and one region on each of GGA8, GGA9, GGA15, GGA18, and GGA28 (Table 4 and Table S5).

### 3.5. Identification of Key Candidate Genes Affected by Selection

As a result of sieving and examining the genomic regions of selective sweeps determined by at least one method, we structurally annotated these regions and revealed the presence of 881 candidate genes in the two studied chicken populations (Table 5 and Table S6). Those involved 548 annotated genes, including 524 protein coding genes and 24 micro RNAs, with many of them being previously described in other relevant studies (e.g., *ALX1*, *DIO2*, *GJD2*, *KITLG*, *TSHR*, etc.). The rest of them were 316 novel Ensembl genes, 10 uncharacterized NCBI loci, and 7 uncharacterized chicken homologs to human genes. In all, there were 516 chicken–human orthologs used later for the GO analysis.

Among the discovered 881 candidate genes, 762 genes were found on nine macrochromosomes (GGA1–9) and 119 genes on six microchromosomes (GGA13, GGA14, GGA15, GGA18, GGA26, and GGA28). Total length of presumptive selection footprints identified on macro- and microchromosomes was respectively 46.46 and 3.32 Mb. This candidate gene mining survey resulted in an estimate of ~16.4 genes on an average every 1 Mb of macrochromosomal DNA and ~35.9 genes per 1 Mb on microchromosomes, or a ~2.2-fold difference.

In Table 5, a shorter list is presented for 188 prioritized candidate genes that were annotated in either chicken or human genomes and retrieved from the 11 overlapped regions of putative selection as identified at least by two techniques. These included the following 50 previously known candidate genes (Table 5, shown in bold; see also their details in Table S6): *ALX1*, *ANKRD13C*, *BTG1*, *FGF3*, *FGF4*, *KITLG*, *LEPR*, *MGAT4C, NAT10*, *RPE65*, *SHANK2*, and *TYW3* (identified in the RW breed); *AP1M1*, *AVEN*, *CALR3*, *ELL*, *FMN1*, *GREM1*, *INSR*, *KLF2*, *LOC420160*, *MIR6666*, *MIR6693*, *MYDGF*, *MYO9B*, *PLIN3*, *PTPRS*, *PCDH7*, *RAB8A*, *RABGAP1L*, *RYR3*, *SMIM7*, *SPRED1*, *TICAM1*, *TMEM38A*, and *USE1* (in the WC breed); and *ACTC1*, *BTBD9*, *CCT5*, *CMBL*, *CTNND2*, *DAP*, *FASTKD3*, *GLP1R*, *GJD2*, *MEIS2*, *MTRR*, *ROPN1L*, *SEMA5A*, and *SRD5A1* (in both the RW and WC breeds).

Many other key candidate genes, including those suggested in relevant studies (e.g., *ANKH*, *ANXA10*, *BMPR1B*, *CASP6*, *CD36*, *CHST11*, *COL6A1*, *COL6A2*, *CRY1*, *DHX36*, *DIO2*, *FGA*, *FGB*, *FGG*, *GTF2A1*, *IGF1*, *IGSF10*, *MMP16*, *MYF5*, *MYF6*, *NUAK1*, *OVST*, *PLRG1*, *PMCH*, *SFRP2*, *SH3RF2*, *SLC2A14*, *SPOCK3*, *TSHR*, *TMEM263*, *WWP1*, etc.) were determined in other genomic regions with selective sweeps in the RW and/or WC chickens using either method (Tables S5 and S6).

### 3.6. QTLs Overlapped with the Identified Genomic Regions

Using Chicken QTLdb [68], we completed a search that showed the presence of 198 known QTLs overlapped with 45 identified genomic regions localized on GGA1 (six regions), GGA2 (five regions), GGA3 (five regions), GGA4 (seven regions), GGA5 (three regions), GGA6 (one region), GGA7 (five regions), GGA8 (two regions), GGA9 (three regions), GGA13 (one region), GGA14 (one region), GGA15 (one region), GGA18 (two regions), GGA26 (one region), and GGA28 (two regions) (Table 6 and Table S7).

The retrieved QTL regions also overlapped with the following five associated genes: *CRY1*, *CHST11*, *TMEM263*, and *NUAK1* on GGA1, and *BMPR1B* on GGA4 (Table 6 and Table S7).

### 3.7. Functional Annotation and GO Term Enrichment

Using the DAVID web tool and a list of 516 chicken–human orthologous genes found in the genomic regions with selection signatures (Table S6), we performed the analysis of functional annotation and enrichment of GO terms. A number of key candidate genes underlying the selective sweeps were determined as summarized in Table 7.

## 4. Discussion

### 4.1. Genetic Diversity and Evolutionary Relationships

In the current study, we have taken advantage of examining and comparing two breeds that are typical representatives of two major, different, and, in a certain sense, opposite evolutionary lineages occurred in the process of chicken domestication and breeding [9]. That is, one was selected for egg-type traits (RW) and the other for meat-type traits (WC). We found that 15.2% of variability was caused by genetic differences between these two studied breeds (*F*_ST_ = 0.152), and the remaining 84.8% was due to allelic variation within the breeds. We observed a significantly lower level of genetic diversity in RW chickens as compared to WC (*_U_H_E_* = 0.339 vs. 0.383) (Table 1). A possible reason for this can be the genetic drift occurred in the population of RW chickens, which has a very small size (~260 heads) and has been subjected to a long-term breeding as a closed population. At the same time, a higher level of genetic diversity in WC chickens may also reflect a crossbred origin of the individuals used in the present study. A higher value of *F*_ROH_ inbreeding coefficient, which was observed in RW chickens (0.195 vs. 0.172) (Table 2), may reflect the origin of the studied RW population from a small number of founders [11]. A slight excess of heterozygotes observed in the RW population may be a consequence of a lower selection pressure and selection for a variety of breeding traits [15,18] as compared to WC [23]. Genetic drift can be considered another possible reason that led to a significant deviation in the number of heterozygotes among RW chickens as compared to that expected under Hardy–Weinberg equilibrium.

The results of the PCA plotting, Neighbor-Net analysis, and admixture clustering (Figure 2a–c) clearly distinguished the RW and WC breeds, confirming their different genetic origin.

As the egg-type breed, the RW chickens, exposed also in the past to crossing with a typical egg layer breed of White Leghorns, were selected for fecundity, egg number, and other egg performance traits. In contrast, the WC breed was derived from crosses between meat and game breeds and selected for growth, muscle development, and other meat production traits. Both breeds manifesting different phenotypic traits were previously included by Moiseyeva et al. [9] in a detailed comparative phylogenetic survey of various chicken breeds using two sets of morphological discrete characters, body measurements, biochemical markers, and the activity of serum esterase-1 (currently, CES1L1, carboxylesterase 1 like 1). In the generated dendrograms for evolutionary relationships in chickens, RW clustered with the White Leghorn and other egg-type breeds, whereas WC formed common clusters with game and meat-type breeds. Lately, using the Illumina Chicken 60K SNP iSelect BeadChip, Dementieva et al. [17] localized RW and WC on the opposite branches of an *F*_ST_-based Neighbor Joining tree. Our data based on Chicken 50K_CobbCons chip-assisted SNP genotypes strongly supported these previous phylogenetic relationship assessments for the RW and WC breeds that exemplify typical egg- and meat-type chickens.

### 4.2. Genomic Trajectories of Selection

To the best of our knowledge, this is the first study of putative signatures of selection in the genomes of the native egg-type RW breed (Figure 1a). We performed it based on the analysis of genome-wide SNP genotypes using 44,728 autosomal SNPs. In the time of the Soviet Union, RW was the major layer breed used for egg production [10]. Currently, only a small number of cold-tolerant and disease-resistant RW chickens are kept in the gene pool collection [19,20]. Identifying genomic regions affected by natural and artificial selection in breeds with different genetic backgrounds may give an insight into the history of domestication and selection for economically important traits [76,77,78,79]. Therefore, we chose for comparison the meat-type WC breed, which is characterized by high growth rate and outstanding meat productivity [23]. We believe that the breed-specific genomic regions found as being under selection pressure are most likely related to recent artificial selection and harbor genes and their variants associated with breeding traits. On the contrary, the regions, which overlap in the two breeds that manifest contrasting phenotypic traits, may reflect more ancient evolution events prior to domestication or breed specialization.

Three different statistics (selecting top 0.1% SNPs by *F*_ST_ value, hapFLK analysis, and detection of ROH islands shared by more than 50% of individuals) were applied to detect the genomic regions and genes that are affected by selection in RW and WC chickens (Table 3, Table 4 and Table 5, Tables S5 and S6). Using multiple methods based on different approaches may complement each other and therefore have higher informative power [35,80]. Among 45 SNPs that were selected by *F*_ST_ value (Table S2), 33 were localized within the regions identified by hapFLK or ROH analysis (Table S5). Among 15 genomic regions identified by hapFLK analysis, five were overlapped with ROH islands (Table 3). In total, we identified 11 true genomic regions under selection pressure (so were identified by at least two different methods), including four RW-specific selection sweeps on GGA1, GGA5, GGA8, and GGA9; four WC-specific signatures of selection on GGA1, GGA5, GGA8, and GGA28; and three regions on GGA2 (two sweeps) and GGA3, which were common for both breeds (Table 3). Comparing selection signatures identified in the present study with the results of previous investigations performed in the RW, WC, and other chicken breeds that support our findings [15,21,38,39,40,41,72,73,74,75], we showed the presence of 31 overlapped genomic regions distributed among twelve chromosomes (Table 4 and Table S5).

### 4.3. Key Candidate Genes and Overlapping QTLs within Sweep Regions

The present study provided a rich estimation of coding genes that underlie certain regions subject to selection in the chicken genome. Of especial interest are those that formed a set of 188 prioritized candidate genes as detected by more than two approaches to hunting for selection signatures in the genomes of the two studied breeds with contrasting phenotypes (Table 5). Of particular note are the genes ascertained in other genome-wide or more focused investigations targeting selection footprints, ROHs, QTLs, and candidate genes associated with important phenotypes and functions (shown in Table 5 in bold). Among them, we emphasized ten genes specific to the egg-type RW breed sweep regions. For the meat-type WC breed regions, there were 24 specific genes. In both breeds, we observed the following 14 candidates: *ACTC1*, *BTBD9*, *CCT5*, *CMBL*, *CTNND2*, *DAP*, *FASTKD3*, *GLP1R*, *GJD2*, *MEIS2*, *MTRR*, *ROPN1L*, *SEMA5A*, and *SRD5A1*. We outline these genes below in a more detail-oriented manner, along with characterizing the appropriate QTLs known within the same regions.

#### 4.3.1. GGA1 Region Candidate Genes and QTLs

The *ALX1* gene (41,898,277 … 41,919,541 bp), a member of the aristaless-like homeobox (ALX) family, is associated with craniofacial and limb development [81]. In chickens, the gene is involved in beak morphology and was shown to have been under selection prior to domestication, being colocalized with a selective sweep on GGA1 [40], i.e., similarly to our finding. Within the same genomic region, there are three other genes, *BTG1* (44,429,339 … 44,433,309 bp)*, KITLG* (43,015,486 … 43,066,975 bp), and *MGAT4C* (42,251,047 … 42,358,204 bp). BTG anti-proliferation factor 1 (*BTG1*) is a candidate gene expressed during early chick development [82], being related to muscle structure growth and development at early stages [83]. The *KITLG* (ligand of tyrosine–kinase receptor) gene was cytogenetically mapped to GGA1 [84] and more recently ascertained as a gene related to pigmentation traits, having been under selection before chicken domestication and overlapping a selective sweep [40]. *MGAT4C* (MGAT4 family member C) was recognized as a differentially expressed gene associated with nonsynonymous SNPs and putative selective signaling, suggesting its relation to chicken adaptation to high-altitude conditions [85].

Six selective sweeps (two RW-specific and four WC-specific) identified on GGA1 were overlapped with 28 known QTLs (Table S7). The region of 42.2 … 44.5 Mb under selection in RW chickens was associated with multiple QTLs for egg yolk weight (QTL:24938, QTL:24939, and QTL:24940; within 42,044,310 … 42,991,116 bp) that might be a result of recent selection for increased egg weight. Selective sweeps found in WC chickens mainly covered QTLs for growth, carcass traits, and feed efficiency including body weight at 9 days (QTL:96626, 45,501,491 … 54,773,135 bp), feed conversion ratio (QTL:139668, 54,874,224 … 54,874,264 bp; and QTL:139747, 76,365,781 … 76,365,821 bp), abdominal fat weight (QTL:19362553, 914,126 … 55,103,802 bp; and QTL:193624, 52,797,908 … 53,910,398 bp), and carcass fat content (QTL:193637, 52,797,908 … 53,910,398 bp) (Table 6 and Table S7).

#### 4.3.2. GGA2 Region Candidate Genes and QTLs

Previously, Qanbari et al. [38] detected signals of selective sweeps in this region embracing candidate genes in laying hens that might be under selection pressure. In our study, we identified a number of these genes including *CCT5* (chaperonin-containing TCP1 subunit 5; 78,405,510 … 78,413,636 bp), *CMBL* (carboxymethylenebutenolidase homolog; 78,327,060 … 78,338,570 bp), *DAP* (death-associated protein; 78,125,211 … 78,169,242 bp), *FASTKD3* (FAST kinase domains 3; 79,210,863 … 79,217,888 bp), and *ROPN1L* (rhophilin associated tail protein 1 like; 78,263,682 … 78,272,601 bp). Additionally, we established that this region contains some other important genes such as *CTNND2* (77,868,375 … 77,907,969 bp), *MTRR* (79,184,684 … 79,211,008 bp), *SEMA5A* (78,655,148 … 78,914,889 bp), and *SRD5A1*. In another study [86], the *CTNND2* (catenin delta 2; 79,772,665 … 79,786,504 bp) gene was shown to have an extremely strong sweeping signal, with highest zFst score in chromosome 2, though with no relationship to muscle development, growth, or other economic traits. This gene was suggested to be responsible for the evolutionary changes of domestic chickens associated with vision deterioration [86]. *MTRR* (5-methyltetrahydrofolate-homocysteine methyltransferase reductase) is a SNP-containing candidate gene for dermatological diseases/conditions, being also associated with amino acid changes [87]. The gene was also found to be expressed in liver of growing broilers after in ovo injection of folic acid [88]. *SEMA5A* (semaphorin 5A) is a strong candidate gene with selective sweep that is responsible for response to selection on antibody response to sheep red blood cells [73]. In the *SRD5A1* (steroid-5-alpha-reductase, alpha polypeptide 1) gene, a differentially methylated region was found as the most likely biomarker of inbreeding depression of reproduction in Langshan chickens [89,90].

In total, we identified five selective sweeps on GGA2 overlapped with nine known QTLs. Among them, two were specific for RW, one for WC, and two were found in both studied breeds (Table 6 and Table S7). Interestingly, the genomic region within 123.0 … 123.7 Mb identified in RW chickens was overlapped with a previously detected QTL for body temperature (QTL:30853, 121,250,696 … 123,202,074 bp), which could reflect the long-term selection of RW chickens for cold tolerance at the earlier stage of breed development [11,12,13]. Moreover, this region contains a causal SNP associated with day-old chick down color in RW chickens [15].

#### 4.3.3. GGA3 Region Candidate Genes and QTLs

Of a particular interest within this region are two candidates for the development and weight of the comb, *BTBD9* (BTB domain containing 9; 13,189,289 … 13,199,549 bp) and *GLP1R* (glucagon like peptide 1 receptor; 29,410,438 … 29,492,850 bp) [91]. For the latter gene, a signal of selective sweeps was also determined by Qanbari et al. [40].

Among five selective sweeps overlapped with 16 known QTLs, three were specific for the RW breed, one for WC, and two were common for both studied breeds (Table 6 and Table S7). Identification of additional regions in WC chickens (12.8 … 13.5 and 29.2 … 29.8 Mb) associated with QTLs for feed conversion ratio (QTL:139401, 13,278,977 … 13,279,017 bp; and QTL:139333, 29,681,999 … 29,682,039 bp, respectively) suggested that genes associated with feed efficiency could be involved in the artificial selection.

#### 4.3.4. GGA4 Region Candidate Genes and QTLs

The region contains the *PCDH7* (protocadherin 7; 71,575,850 … 71,827,560 bp) gene, a notable positional candidate associated with internal organ traits in chickens and located within a QTL for intestine length and gizzard weight [92] (Table S7).

We also found seven selective sweeps in the genomes of RW and WC chickens (five RW-specific and two WC-specific), which covered 31 previously described QTLs (Table 6 and Table S7). A selective sweep within 38.0 … 38.6 Mb identified in the genome of RW chickens was overlapped with multiple QTLs for feed intake (QTL:195036 to QTL:195049, QTL:195085, QTL:195087, and QTL:195096). This observation could reflect a selective advantage of RW chickens with high eating capacity that would be able to accumulate more energy necessary to survive at low-temperature environment and maintain a high egg productivity.

#### 4.3.5. GGA5 Region I Candidate Genes and QTLs

A genomic region on GGA5 within 17.8 … 18.8 Mb, which was found to be under selection pressure in RW chickens (Table S5), contains two closely located genes, *FGF3* and *FGF4* (fibroblast growth factors 3 and 4; 17,872,481 … 17,877,619 and 17,843,044 … 17,845,980 bp, respectively), suggested as candidates for the feathered-leg trait [93]. *NAT10* (N-acetyltransferase 10; 18,749,501 … 18,771,124 bp), an additional known candidate gene in laying hens, displays a signal of selective sweeps [38] and contributes to feed conversion ratio [94]. One more gene, *SHANK2* (SH3 and multiple ankyrin repeat domains 2; 18,179,810 … 18,393,764 bp), was localized within a QTL associated with metabolizable efficiency traits in broilers [75].

#### 4.3.6. GGA5 Region II Candidate Genes and QTLs

This region covers 29.6 … 32.9 Mb and comprises a series of vital genes we identified in both the RW and WC chickens. In particular, *ACTC1* (actin alpha cardiac muscle 1; 32,480,478 … 32,485,436 bp) expressed in heart and skeletal muscle was identified as a candidate gene in laying hens that possesses a signal of selective sweeps [38] and is differentially expressed in breast muscle of growing chickens [95]. The *GJD2* (gap junction protein delta 2; 32,502,596 … 32,505,180 bp) gene also has a signal of sweeps, being expressed in brain and retina and potentially involved in adaptation process during chicken domestication [41,96]. An overlapping with a signal of selective sweeps was also found at *RYR3* (ryanodine receptor 3; 30,373,917 … 30,541,375 bp), a supposedly candidate gene for pigment synthesis [97] that is expressed in broiler breast meat in response to heat stress and methionine dipeptide-deficient diet [98].

Interestingly, the region we described here overlaps with ROH islands that were shared between commercial lines and red junglefowls [41] and contain, among other genes, *AVEN* (apoptosis and caspase activation inhibitor; 30,287,850 … 30,373,018 bp), *FMN1* (formin 1; 30,598,320 … 30,751,690 bp), *GREM1* (gremlin 1, DAN family BMP antagonist; 30,776,671 … 30,777,369 bp), *MEIS2* (Meis homeobox 2; 31,437,974 … 31,606,534 bp), and *SPRED1* (sprouty-related EVH1 domain containing 1; 30,948,808 … 31,002,826 bp). Both *AVEN* expressed in adult brain, heart, intestine, kidney, lung, stomach, and spleen, as well as in whole embryos [99], and *GREM1* negatively regulate apoptosis [41]. *GREM1* and *FMN1* play an important role in mouse and chick limb development [100], with the former being located within a chicken QTL related to limb development [41]. *MEIS2* is related to angiogenesis at tibial lesions in broiler chickens [101] and overlapped with a QTL for body weight [41] (Table S7).

#### 4.3.7. GGA8 Region I Candidate Genes and QTLs

Among the genes we localized within this region (7.1 … 7.8 Mb) in the WC genome, *RABGAP1L* (RAB GTPase activating protein 1 like) was lately suggested as a chicken candidate gene for plasma very low-density lipoprotein concentration in a respective GWAS carried out by Zhang et al. [102]. This blood characteristic is thought to be useful for selecting lean meat-type lines [102].

The region found as affected by selection in WC chickens also overlaps with several QTLs associated with feed conversion ratio (QTL:139410; 7,689,799 … 7,689,839 bp), breast muscle pH (QTL:157180; 7,219,671 … 7,569,140 bp), shank circumference (QTL:213550; 7,521,372 … 7,569,140 bp), VLDL cholesterol level (QTL:170802; 7,550,094 … 7,550,134 bp), and antibody titer to IBV (QTL: 24340; 7,500,998 … 7,501,038 bp) (Table S7).

#### 4.3.8. GGA8 Region II Candidate genes

This region within 28.6 … 30.1 Mb that we determined in the RW genome includes few genes of interest, e.g., *LEPR* (leptin receptor; 28,687,054 … 28,717,255 bp), a candidate gene in laying hens harboring a signal of selective sweeps [38]. As a candidate gene suggestive of production-oriented selection [40], it is also associated with growth and feed efficiency in meat-type chickens [103]. Another selection sweep in this candidate region was defined at the *TYW3* (tRNA-yW synthesizing protein 3 homolog; 29,988,144 … 29,991,956 bp) gene [104]. *ANKRD13C* (ankyrin repeat domain 13C; 29,389,240 … 29,401,259 bp) gene was suggested to be a candidate gene for feed conversion ratio [94]. The *RPE65* (retinoid isomerohydrolase; 29,124,295 … 29,130,623 bp) gene is related to retinol metabolism, its expression being downregulated in rose-comb chickens [105].

#### 4.3.9. GGA28 Region Candidate Genes and QTLs

We identified within this region (4.0 … 5.0 Mb) several other recently suggested candidate genes for plasma very low-density lipoprotein concentration as determined by Zhang et al. [102], including *AP1M1* (adaptor-related protein complex 1 mu 1 subunit; 4,510,264 … 4,518,819 bp)*, CALR3* (calreticulin 3; 4,437,755 … 4,441,529 bp)*, ELL* (elongation factor for RNA polymerase II; 3,997,087 … 4,031,848 bp)*, KLF2* (Kruppel-like factor 2; 4,484,819 … 4,487,194 bp)*, MIR6666* (microRNA 6666; 4,808,669 … 4,808,772 bp)*, MIR6693* (microRNA 6693; 4,387,579 … 4,387,688 bp)*, MYO9B* (myosin IXB; 4,263,083 … 4,303,548 bp)*, PTPRS* (protein tyrosine phosphatase, receptor type S; 4,727,381 … 4,835,713 bp)*, RAB8A* (RAB8A, member RAS oncogene family; 4,524,693 … 4,537,499 bp)*, SMIM7* (small integral membrane protein 7; 4,391,143 … 4,393,274 bp)*, TICAM1* (toll-like receptor adaptor molecule 1; 4,961,810 … 4,965,486 bp)*, TMEM38A* (transmembrane protein 38A; 4,384,819 … 4,391,056 bp)*,* and *USE1* (unconventional SNARE in the ER 1; 4,258,017 … 4,262,921 bp). *AP1M1* is involved in endosome to melanosome transport [106] and *USE1* [38] is overlapped with signals of selective sweeps. *ELL* is also a positional candidate gene for egg number in broiler breeders [107] and a candidate gene associated with egg quality (yolk weight) [108]. The *KLF2* gene is related to angiogenesis at tibial lesions in broiler chickens [101]. Our findings extend knowledge about genes under selection within this region on GGA28 in meat-type chickens.

In addition, this region embraced few other important genes as demonstrated in our and other investigations. *INSR* (insulin receptor; 4,218,437 … 4,253,117 bp) is known as a candidate gene in laying hens that contains a signal of selective sweeps [38]. *LOC420160* (cathepsin L1-like; 5,030,693 … 5,034,171 bp) is a chicken ortholog to human *CTSV* (cathepsin V) and *CTSL* (cathepsin L), cysteine cathepsins being attributed to extracellular matrix degradation and tissue remodeling [109]. Initially, the chicken *CTSL* (now *CTSV*) gene that has an increased expression during oviduct regression [110] was mapped to the Z chromosome [111], while *LOC420160* is considered as its paralog on GGA28. *MYDGF* (myeloid-derived growth factor; 5,034,853 … 5,039,077 bp) is a candidate gene related to growth and development at early stages [83]. The *PLIN3* (perilipin 3; 4,952,051 … 4,961,161 bp) gene is associated with immunity and enhances growth performance in broilers [112].

The region selected in WC chickens overlapped with several previously described QTLs associated with fat content (QTL:193631 for abdominal fat content; QTL:193655 and QTL:193647 for carcass fat content; within 3,753,016 … 4,712,620 bp) and blood parameters including blood pH (QTL:71128; 3,856,132 … 4,097,788 bp), CO2 partial pressure (QTL:71133 and QTL:71137; 3,835,952 … 4,167,579 bp), mean blood cell volume (QTL:71178; 3,944,019 … 4,197,143 bp), and blood hemoglobin level (QTL:71183; 3,944,019 … 4,197,143 bp) (Table S7).

#### 4.3.10. Other Important Candidate Genes

Our genome-wide screening resulted in establishing multiple candidate genes underlying the sweep regions and potentially affected by selection for egg and meat production traits in the genomes of the egg-type RW and meat-type WC chickens as identified by either technique (Tables S5 and S6). Many of them were supported by other previous studies and deserve a further attention. For instance, Qanbari et al. [38,40] reported signals of selective sweeps for the following candidate genes in laying hens (with respective chromosome of their location indicated in the parentheses): *ANKH* (ANKH inorganic pyrophosphate transport regulator; GGA1), *IGF1* (insulin-like growth factor 1; GGA1), *BMPR1B* (bone morphogenetic protein receptor type 1B; GGA4), *TSHR* (thyroid stimulating hormone receptor; GGA5), and *IGSF10* (immunoglobulin superfamily member 10; GGA9). Among these, *IGF1* is also an important growth factor [40] associated with abdominal fat weight/deposition, body weight, and other traits in chickens [113,114], and *BMPR1B* is a candidate gene for low methylation related to hypoxic adaptation in Tibetan chickens [115]. The *BMPR1B* gene was also found within a feed intake-associated QTL [68] (Table 6).

On GGA1, we defined a few more candidates under selection in the egg-type breed. In particular, signals of selective sweeps were observed at the *CD36* (CD36 molecule) gene [116] and at the closely located *MYF5* and *MYF6* (myogenic factors 5 and 6) genes [106]. *CD36* is a candidate gene for pendulous comb [116] and related to adipogenesis and lipogenesis in pectoralis muscle tissue [117]. In the meat-type breed, signals of selective sweeps overlapped with *OVST* (ovostatin) [118], *PMCH* (promelanin concentrating hormone) [37,119], and *SLC2A14* (solute carrier family 2 member 14) [120]. The *OVST* gene is involved in oviduct development and eggshell formation [118]. *PMCH* is a candidate gene involved in chicken growth control and overexpressed in low growth rate broiler cockerels [119] that also contributes to appetite and food intake [37,118]. *SLC2A14* is supposedly a candidate gene for pigment synthesis [120].

Among other candidates, we also discovered on GGA2 that there is the *WWP1* (WW domain containing E3 ubiquitin protein ligase 1) gene, a selection candidate for muscularity in broilers detected as signal of parallel divergence [96]. Quite close to this gene, Kudinov et al. [15] found a SNP rs15151359 that was one of eight markers suggestively associated in RW chickens with the snow-white down color at day-old. This SNP is flanked by the *MMP16* (matrix metallopeptidase 16) gene [15], although the latter overlaps with a novel gene *ENSGALG00000053033* (Table S6) that is homologous to RNA-directed DNA polymerase from mobile element jockey-like. We identified *MMP16* and *ENSGALG00000053033* in the RW-specific selective sweep region. Because we have no other relevant information about these two genes in chickens, further speculation about their contribution to phenotypic traits in the RW breed would require an additional investigation.

Within a GGA4-specific ROH, we identified *ANXA10* (annexin A10), a candidate gene for abdominal fat in a copy number variation (CNV) region overlapping with a QTL and a selective sweep [121]. Among other genes in RW-specific regions under putative selection on GGA4, there was a trio of the linked fibrinogen alpha, beta, and gamma chain genes (*FGA*, *FGB*, *FGG*) that are involved in cell differentiation and had signals of selective sweeps [106]. *FGA* is also related to response to Marek’s disease [122], while *FGB* is a candidate gene overexpressed in low growth rate broiler cockerels [119]. Two more closely located genes, *PLRG1* (pleiotropic regulator 1) and *SFRP2* (secreted frizzled related protein 2), also overlap with signals of selective sweeps and contribute to cell differentiation. *SFRP2* is involved in inbreeding depression of reproduction [90] and embryogenesis (development of the neural system, eyes, muscles, and limbs) [123], and was described as a candidate gene for adaptation to solar radiation in North- and East-African chickens [106]. *SPOCK3* (SPARC/osteonectin, cwcv, and kazal-like domains proteoglycan 3) is a candidate gene for abdominal fat within a CNV region overlapped with a QTL and a selective sweep [121]. An ROH island on GGA4 found in the WC genome harbored the *CASP6* (caspase 6) gene that is involved in regulation of apoptosis and was reported to be upregulated in broiler chickens in response to *Salmonella* and phytobiotic intake [124].

Most intriguingly, we identified a ROH on GGA5 in the egg-type RW breed that involves the *TSHR* gene related to reproductive machinery and most prominent for being coined as a domestication gene in chickens [37,40,118,125]. Moreover, we also confirmed the known candidate genes located within the same LD block as *TSHR*, such as *CEP128* (centrosomal protein 128), *DIO2* (deiodinase, iodothyronine type II), *GTF2A1* (general transcription factor IIA subunit 1), *SEL1L* (SEL1L adaptor subunit of ERAD E3 ubiquitin ligase), and *STON2* (stonin 2) [126]. *DIO2* is a candidate gene, overexpressed in low growth rate broiler cockerels [120], involved in regulation of hormone levels and possibly affected by the domesticated mutation at *TSHR* [126]. *GTF2A1* is a candidate gene for egg production traits [127] colocalized with a signal of selective sweeps [104]. The *SEL1L* and *STON2* genes are candidates associated with egg number in laying hens [127], and *STON2* is also a candidate gene related to yolk weight [108].

Next, we detected a ROH on GGA7 in the meat-type WC breed that contains two notable genes, *COL6A1* and *COL6A2* (collagen type VI alpha 1 chain and collagen type VI alpha 2 chain). These are candidate genes involved in skeletal system development and overexpressed in low growth rate broiler cockerels [120]. They also contribute to meat quality and bear strong selection signals in Chinese indigenous chicken genomes [97]. Through the DNA methylation mechanism, the *COL6A1* gene modification affects intramuscular fat deposition in chicken [128] and relates to meat quality of breast muscle [129]. The *COL6A2* gene was also listed among candidates for feed conversion ratio [94].

Two more genes are noteworthy to discuss here: *DHX36* (DEAH-box helicase 36) and *SH3RF2* (SH3 domain containing ring finger 2). The *DHX36* gene is located on GGA9 within breakpoint of evolutionary conservation between human and chicken chromosomes [130] and serves as an RNA sensor in innate immunity for recognizing viral RNA [131]. Within a WC-specific genomic region on GGA13, we also determined *SH3RF2*, a strong candidate gene in broilers that has a signal of selective sweeps and gene deletion associated with increased growth, while lying within a QTL region for body weight [37].

### 4.4. GO Term Annotation Clustering of Candidate Genes

As a result of GO analysis, we found four significant annotation clusters enriched with GO terms that underlay key candidate genes of interest derived from the regions of selection footprints in the RW and WC breeds. Remarkably, these GO term clusters involved certain important genes, most of which we have outlined above, e.g., *WWP1*, *IGF1*, *INSR*, *SEMA5A*, *RPE65*, and *COL6A2*. A couple of noteworthy candidates can also be mentioned here, including *PIK3CB* (phosphatidylinositol-4,5-bisphosphate 3-kinase catalytic subunit beta) and *TGFBI* (transforming growth factor beta-induced). The *PIK3CB* gene is associated with white/red earlobe color formation in chicken [132]. The *TGFBI* gene is related to angiogenesis at tibial lesions in broiler chickens [102] and was reported to be a candidate gene for Müllerian duct development and its disorders [133].

### 4.5. Gene Richness

In terms of comparing gene density on chicken macro- and microchromosomes, we found that microchromosomes contained 2.2 times more genes in the genomic regions harboring selection signatures. This observation is highly concordant with the previous general estimates of two- to three-fold gene richness on microchromosomes as compared to that on macrochromosomes (e.g., [134]), demonstrating a clear-cut negative correlation between gene density and chromosome length in the chicken genome [8].

## 5. Conclusions

Using three complementary statistical methods (*F*_ST_ at pairwise breed comparison, hapFLK analysis, and identification of ROH islands), we performed the search for genomic footprints in two contrasting chicken breeds, the native egg-type RW and meat-type WC. Eleven true genomic regions under selection pressure were identified by at least two different methods as distributed on seven *G. gallus* autosomes. Four of such selective sweep regions were breed-specific for each of RW and WC chickens, while three remaining regions were common for both breeds. Several identified genomic regions were overlapped with known QTLs. In RW chickens, these regions included known QTLs for body temperature, egg production rate, egg yolk weight, and feed intake that are in accordance with the breed origin and the history of its artificial selection for cold tolerance and egg laying. Selective sweeps identified in the genome of WC chickens were mainly overlapped with QTLs responsible for growth, meat and carcass traits, and feed conversion ratio that can reflect the long-term selection of this breed for increased growth rate, meat productivity, and feed efficiency. We determined a set of 188 prioritized candidate genes localized within selected genomic regions and reviewed their functional relevance in both breeds. One of RW-specific sweep regions contained the *TSHR* gene known for being associated with domestication and reproduction in chickens. Examination of gene ontology in the sweep regions added more information for their functional annotation in two breeds. We also suggested a reasonable estimate of greater candidate gene richness within sweep regions on microchromosomes vs. macrochromosomes. These findings extend our knowledge about genomic diversity and genes under selection in the genomes of two chicken breeds with different selection histories and contrasting phenotypic traits. The research results will be useful for conservation, sustainable breeding, and efficient selection of the Russian White chicken breed.

## Figures and Tables

**Table 1:** Summary of genetic/genomic diversity statistics ^1^ calculated in the studied Russian White (RW) and White Cornish (WC) breeds based on SNP genotypes.

Breed	*n*	*H_O_* (M ± SE)	*_U_H_E_* (M ± SE)	*_U_F*_IS_ [CI 95%]	*A_R_* (M ± SE)
RW	31	0.345 ± 0.001	0.339 ± 0.001	–0.016 [–0.018; –0.014]	1.937 ± 0.001
WC	23	0.380 ± 0.001	0.383 ± 0.001	0.009 [0.006; 0.012]	1.982 ± 0.001

^1^ *n*, number of individuals; *H_O_*, observed heterozygosity; M, mean value; SE, standard error; *_U_H_E_*, unbiased expected heterozygosity; *_U_F*_IS_, unbiased inbreeding coefficient (CI 95%, range variation of *_U_F*_IS_ coefficient at a confidence interval of 95%); *A_R_*, rarefied allelic richness [47].

**Table 2:** Summary of the runs of homozygosity (ROH) descriptive statistics ^1^ calculated in the studied Russian White (RW) and White Cornish (WC) breeds based on SNP genotypes.

Breed	*n*	ROH No.			ROH Length, Mb			*F*_ROH_ (M ± SE)
		(M ± SE)	Min	Max	(M ± SE)	Min	Max	
RW	31	109.10 ± 3.08	62	149	183.41 ± 10.63	50.92	325.96	0.195 ± 0.011
WC	23	119.09 ± 2.44	100	144	161.97 ± 5.09	91.49	190.61	0.172 ± 0.005

^1^ *n*, number of individuals; ROH No., the number of ROHs in a genome; ROH Length, the overall length of ROHs in a genome; *F*_ROH_, inbreeding coefficient calculated based on ROHs; M, mean value; SE, standard error; min, minimal value; and max, maximal value.

**Table 3:** Overlapped genomic regions under putative selection identified at least by two different statistics in the Russian White (RW) and White Cornish (WC) breeds.

GGA ^1^	Genomic Regions (Bp) ^2^ under Selection Identified by Different Methods		
	*F*_ST_^3,6^	*hapFLK* ^4^	*ROH50* ^5^
1		41,095,175 … 44,484,517 ^7^	41,095,175 … 41,733,094 ^7^ 41,955,503 … 43,335,920 ^7^
2		69,808,276 … 72,600,534 ^7,8^	69,624,561 … 70,806,756 ^8^ 71,053,537 … 73,559,354 ^8^
2	79,243,261; 79,256,813	76,552,199 … 80,316,420 ^7,8^	78,172,432 … 78,579,336 ^8^ 78,935,731 … 79,963,158 ^8^ 79,022,396 … 79,487,897 ^7^
3	29,676,636	29,203,776 … 29,759,199 ^7,8^	28,963,634 … 29,993,389 ^7^
4	70,173,798; 71,530,773		70,850,437 … 71,917,421 ^8^
5	18,592,354		17,793,200 … 18,750,396 ^7^
5	30,348,807, 31,277,206 … 32,275,187	31,103,041 … 32,810,342 ^8^	29,655,633 … 32,895,623 ^8^
8	7,120,493 … 7,373,397		7,120,493 … 7,750,507 ^8^
8		28,777,070 … 30,174,896 ^7^	28,633,681 … 29,051,709 ^7^
9	17,770,251; 17,880,381		17,985,436 … 18,286,878 ^7^
28	4,011,131; 4,419,938		4,240,969 … 5,038,396 ^8^

^1^ GGA, *Gallus gallus* autosomes. ^2^ Genomic regions: start and end positions (bp) according to the GGA reference genome assembly GRCg6a [45]. Methods used for defining signatures of selection: ^3^ *F*_ST_, top 0.1% SNPs by *F*_ST_ value at pairwise population comparison; ^4^ *hapFLK*, regions identified by hapFLK analysis at *p* < 0.01; and ^5^ *ROH50*, ROH segments distributed in more than 50% of animals within each breed. Studied populations: ^6^ RW/WC comparison, ^7^ RW, and ^8^ WC.

**Table 4:** Overlapped genomic regions identified in the present and previous studies.

#	GGA ^1^	Present Study			Previous Studies			
		Method ^2^	Region/SNP Position ^3^	Breed/Population ^4^	Method ^2^	Region/SNP Position ^3^	Breed/Population ^4^	Citation
1	1	ROH	53.44 … 55.59	WC	*wF*_ST_	49.37 … 54.56	VAL	[73]
					XP-EHH, XP-CLR	54.77 … 55.06	WC-ML3, WR-FL2	[72]
					*H_P_*	55.28 … 55.33	BL	[38]
					*wF*_ST_	55.33 … 55.37	RJF/Coms	[40]
2	1	ROH	143.05 … 143.93	RW	*wF*_ST_	142.51 … 144.83	VAL	[73]
						143.46 … 143.50	RJF/Coms	[40]
			143.27 … 144.07	WC		142.51 … 144.83	VAL	[73]
						143.46 … 143.50	RJF/Coms	[40]
3	1	hapFLK	160.61 … 161.56	WC	*wF*_ST_	157.11 … 162.35	VAL	[73]
4	2	ROH	24.86 … 26.03	WC	*wF*_ST_	25.75 … 25.79	RJF/Coms	[40]
					QTL	24.54 … 25.09	broilers	[41]
5	2	*F*_ST_	39.62	RW/WC	*wF*_ST_	34.34 … 42.94	VAL	[73]
6	2	*F*_ST_	67.57	RW/WC	*wF*_ST_	66.93 … 67.93	VAL	[73]
7	2	ROH	69.62 … 70.81	WC	ROH	68.23 … 73.46	RW	[21]
		hapFLK	69.81 … 72.60	RW, WC	*wF*_ST_	72.49 … 73.27	VAL	[73]
		ROH	71.05 … 73.56	WC	ROH	68.23 … 73.46	RW	[21]
					*wF*_ST_	73.06 … 73.10	RJF/Coms	[40]
8	2	ROH	75.48 … 76.22	WC	*H_P_*	75.73 … 79.65	BL	[38]
9	2	hapFLK	76.55 … 80.32	RW, WC	*H_P_*	75.73 … 79.65	BL	[38]
					*F*_ST_	78.19	VAL	[73]
					*wF*_ST_	78.76 … 78.81 79.95 … 80.13		
		ROH	76.55 … 77.20 77.64 … 78.58 78.94 … 79.96	WC	*H_P_*	75.73 … 79.65	BL	[38]
					*F*_ST_	78.19	VAL	[73]
					*wF*_ST_	79.95 … 80.13		
			79.02 … 79.49	RW	*H_P_*	75.73 … 79.65	BL	[38]
		*F*_ST_	79.24 79.26	RW/WC				
10	2	ROH	123.02 … 123.74	RW	*H_P_*	122.25 … 124.67	BL	[38]
					GWAS	123.45	RW	[15]
11	3	ROH	12.80 … 13.45	WC	*F*_ST_	11.56 … 13.00	VAL	[73]
12	3	ROH	28.96 … 29.99	RW	*wF*_ST_	29.44 … 29.48	RJF/Coms	[40]
		hapFLK	29.20 … 29.76	RW, WC				
13	3	ROH	83.31 … 84.12	RW	QTL	83.74 … 84.06	broilers	[41]
14	4	ROH	17.87 … 20.34	RW	*wF*_ST_	16.90 … 21.86	VAL	[73]
					QTL	18.33 … 18.70	RJF	[40]
15	4	ROH	24.23 … 24.99	RW	ROH	22.62 … 28.37	RW	[21]
					*wF*_ST_	24.26 … 26.54	VAL	[73]
16	4	hapFLK	33.48 … 34.34	RW	*wF*_ST_	32.61 … 33.48	VAL	[73]
17	4	ROH	57.73 … 58.38	WC	*H_P_*	57.93 … 58.96	BL	[38]
18	4	*F*_ST_	70.17	RW/WC	*wF*_ST_	68.02 … 73.21	VAL	[73]
		ROH	70.85 … 72.00	WC		71.29 … 71.31	RJF/Coms	[40]
		*F*_ST_	71.53	RW/WC	*wF*_ST_	68.02 … 73.21	VAL	[73]
					*ZHp*	71.37 … 71.60	broiler, BRS	[39]
19	5	ROH	17.79 … 18.75	RW	*H_P_*	17.31 … 19.10	BL	[38]
					*wF*_ST_	17.45 … 18.39	VAL	[73]
					QTL	18.31 … 19.00	broilers	[75]
		*F*_ST_	18.59	RW/WC	*H_P_*	17.31 … 19.10	BL	[38]
					QTL	18.31 … 19.00	broilers	[75]
20	5	ROH	29.66 … 32.90	WC	*wF*_ST_	30.12 … 30.16	RJF/Coms	[40]
					QTL	30.26 … 32.06	broilers	[41]
					*wF*_ST_	30.46 … 31.39	VAL	[73]
						31.30 … 31.34	RJF/Coms	[40]
					XP-EHH, XP-CLR	31.99 … 32.75	WC-ML1, WC-ML3	[72]
					*wF*_ST_	32.50 … 32.54	RJF/Coms	[40]
		*F*_ST_	30.35	RW/WC	QTL	30.26 … 32.06	broilers	[41]
		hapFLK	31.10 … 32.81	RW	*wF*_ST_	30.46 … 31.39	VAL	[73]
						31.30 … 31.34	RJF/Coms	[40]
					XP-EHH, XP-CLR	31.99 … 32.75	WC-ML1, WC-ML3	[72]
					*wF*_ST_	32.50 … 32.54	RJF/Coms	[40]
		*F*_ST_	31.28 … 32.28	RW/WC	QTL	30.26 … 32.06	broilers	[41]
					*wF*_ST_	30.46 … 31.39	VAL	[73]
						31.30 … 31.34	RJF/Coms	[40]
					XP-EHH, XP-CLR	31.99 … 32.75	WC-ML1, WC-ML3	[72]
21	5	ROH	40.50 … 41.21	RW	*F*_ST_, REHH	40.29 … 40.67	Silky fowls	[74]
					*H_P_*	40.97 … 41.02	BL	[38]
22	7	hapFLK	24.18 … 24.20	WC	*wF*_ST_	22.84 … 24.25	VAL	[73]
23	7	ROH	30.27 … 30.84	WC	*wF*_ST_	28.46 … 30.33	VAL	[73]
24	8	ROH	7.12 … 7.75	WC	XP-EHH, XP-CLR	7.31 … 7.44	WC-ML1, WC-ML2, WR-FL1	[72]
		*F*_ST_	7.32 7.34 7.37	RW/WC				
25	8	ROH	28.63 … 29.05	RW	*H_P_*	28.69 … 28.72	BL	[38]
					Δ*AF*	28.72	RJF, Coms	[40]
		hapFLK	28.78 … 30.17	RW	*H_P_*	28.69 … 28.72	BL	[38]
					Δ*AF*	28.72	RJF, Coms	[40]
26	9	hapFLK	23.11 … 24.12	RW	Δ*AF*	23.64	RJF, Coms	[40]
27	13	hapFLK	15.70 … 15.98	RW	*wF*_ST_	15.92 … 17.14	VAL	[73]
28	13	hapFLK	18.25 … 18.88	WC	*F*_ST_, REHH	18.38 … 18.60	dwarf brown-egg layers	[74]
29	15	*F*_ST_	5.63	RW/WC	*wF*_ST_	3.08 … 5.90	VAL	[73]
30	18	ROH	0.02 … 0.83	WC	*wF*_ST_	0.03 … 3.34	VAL	[73]
					XP-EHH, XP-CLR	0.25 … 0.43	WC-ML1, WR-FL1	[72]
31	28	*F*_ST_	4.01 4.42	RW/WC	*wF*_ST_	3.29 … 4.60	VAL	[73]
		ROH	4.24 … 5.04	WC				
					*H_P_*	4.22 … 4.26	BL	[38]

#, designation of an ordinal number. ^1^ GGA, *Gallus gallus* autosomes. ^2^ Methods used for defining signatures of selection: ROH, runs of homozygosity islands shared in more than 50% of individuals; hapFLK, regions identified by hapFLK analysis at *p* < 0.01; *F*_ST_, top 0.1% of SNPs by *F*_ST_ value at pairwise population comparison; *H_P_*, pooled heterozygosity; XP-EHH, cross-population extended haplotype homozygosity; XP-CLR, cross-population composite likelihood ratio; *wF*_ST_, window-based *F*_ST_ at pairwise population comparison; QTL, quantitative trait locus; *ZHp*, z-transformed pooled heterozygosity statistic; GWAS, genome-wide association studies; REHH, relative extended haplotype homozygosity; Δ*AF*, absolute value of allele frequency difference. ^3^ Region/SNP position: start and end positions (in Mbp) of genomic region or SNP position according to the GGA reference genome assembly GRCg6a [45]. ^4^ Breed/population: WC, White Cornish; RW, Russian White; BL, brown layer; WC-ML1, WC-ML2 and WC-ML3, White Cornish male (broiler sire) lines 1, 2, and 3; WR-FL1 and WR-FL2, White Rock female (broiler dam) lines 1 and 2; VAL, Virginia Antibody lines; RJF, red junglefowls; Coms, four commercial lines.

**Table 5:** Prioritized annotated genes within the overlapped genomic regions affected by putative selection in the Russian White (RW) and White Cornish (WC) chicken breeds and localized by two or three methods.

GGA ^1^	Region (Mb)	Population	Methods ^2^	Genes ^3^
1	41.1 … 44.5	RW	ROH, hapFLK	***ALX1***, *ATP2B1*, ***BTG1***, *CEP290*, *DCN*, *DUSP6*, *EPYC*, *KERA*, ***KITLG***, *LUM*, ***MGAT4C***, *NTS*, *POC1B*, *RASSF9*, *SLC6A15*, *TMTC2*, *TMTC3*, *TSPAN19*
2	69.6 … 73.6	WC, RW	ROH, hapFLK	*CDH10*, *CDH9*, *ERV3-1*, *MIR6545*, *PODXL2*, *RNU6-530P*
2	76.6 … 80.3	WC, RW	*F*_ST_, ROH, hapFLK	*ABCA13*, *ADCY2*, *ANKRD33B*, *ATPSCKMT*, ***CCT5***, ***CMBL***, ***CTNND2***, ***DAP***, *DNAH5*, ***FASTKD3***, *MARCHF6*, *MED10*, *MIR1613*, *MIR6562*, ***MTRR***, *NSUN2*, *PAPD7*, *RNU6-383P*, ***ROPN1L***, *SBK2*, ***SEMA5A***, *SNORD123*, *SNRNP48*, ***SRD5A1***, *SUN5*, *TRIO*, *UBE2QL1*, *UPP1*
3	28.9 … 30.0	WC, RW	*F*_ST_, ROH, hapFLK	***BTBD9***, *DAAM2*, *DNAH8*, *GLO1*, ***GLP1R***, *KCNK16*, *KCNK17*, *KCNK5*, *KIF6*, *SAYSD1*, *ZFAND3*
4	70.1 … 72.0	WC	*F*_ST_, ROH	*DTHD1, **PCDH7***
5	17.8 … 18.8	RW	*F*_ST_, ROH	*ACTBL2*, *ANO1*, *CAPRIN1*, *CCND1*, *CD59*, *CTTN*, *FADD*, *FBXO3*, *FGF19*, ***FGF3***, ***FGF4***, *LMO2*, ***NAT10***, *ORAOV1*, *PPFIA1*, ***SHANK2***
5	29.7 … 32.9	WC, RW	*F*_ST_, ROH, hapFLK	***ACTC1***, *AQR*, *ARHGAP11B*, ***AVEN***, *CDIN1*, *CHRM5*, *DPH6*, *EIF2AK4*, *EMC7*, *FAM98B*, ***FMN1***, *FSIP1*, ***GJD2***, *GPR176*, ***GREM1***, *KATNBL1*, ***MEIS2***, *MIR1718*, *MIR6683*, *RASGRP1*, ***RYR3***, *SCG5*, ***SPRED1***, *SRP14*, *STXBP6*, *THBS1*, *ZNF770*
8	7.1 … 7.8	WC	*F*_ST_, ROH	*ASTN1*, *CACYBP*, *LOC112532958*, *MRPS14*, *PAPPA2*, ***RABGAP1L***, *RC3H1*, *RFWD2*, *TNN*, *TNR*
8	28.6 … 30.1	RW	ROH, hapFLK	***ANKRD13C***, *CRYZ*, *CTH*, *DEPDC1*, *DNAJC6*, *ERICH3*, *FPGT*, *GADD45A*, *GNG12*, *IL12RB2*, ***LEPR***, *LEPROT*, *LHX8*, *LOC112532951*, *LOC112532953*, *LRRC7*, *LRRC40*, *MCCC2L*, *MIER1*, *MIR6653*, *MSH4*, *NEGR1*, *PDE4B*, *PTGER3*, *RABGGTB*, ***RPE65***, *SERBP1*, *SGIP1*, *SLC35D1*, *SLC44A5*, *SRSF11*, *TNNI3K*, ***TYW3***, *WLS*, *ZRANB2*
9	17.8 … 18.3	RW	*F*_ST_, ROH	*KCNMB2*, *TBL1XR1*
28	4.0 … 5.0	WC	*F*_ST_, ROH	***AP1M1***, ***CALR3***, *CHERP*, *CIB3*, *CPAMD8*, *DPP9*, ***ELL***, *EPS15L1*, *F2RL3*, *FAM32A*, *FEM1A*, *HAUS8*, ***INSR***, *KDM4B*, ***KLF2***, ***LOC420160***, *MED26*, ***MIR6666***, ***MIR6693***, *MIR7-3*, ***MYDGF***, ***MYO9B***, ***PLIN3***, ***PTPRS***, ***RAB8A***, *SIN3B*, *SLC35E1*, ***SMIM7***, ***TICAM1***, ***TMEM38A***, *TPM4*, *UHRF1*, ***USE1***

^1^ GGA, *Gallus gallus* autosomes. ^2^ Methods used for defining signatures of selection: ROH, runs of homozygosity islands shared in more than 50% of individuals; hapFLK, regions identified by hapFLK analysis at *p* < 0.01; and *F*_ST_, top 0.1% of SNPs by *F*_ST_ value at pairwise population comparison. ^3^ Candidate genes found in other studies are designated in bold (see Table S6 for details).

**Table 6:** Selective sweeps identified in genomes of the Russian White (RW) and White Cornish (WC) chicken breeds, which are overlapped with known QTLs and associated genes.

Trait	GGA	Regions ^1^	Breed	QTL ID ^2^	Associated Genes ^2^
Abdominal fat percentage	3	109.5 … 110.8	RW	14481	
	7	8.3 … 8.8	WC	14504, 14505	
Abdominal fat weight	1	53.4 … 55.6	WC	193625, 193624	
	4	17.9 … 20.3	RW	213534	
	28	4.2 … 5.0	WC	193631	
Aggressive behavior	1	53.4 … 55.6	WC	119901, 119903, 119902, 119893	*CRY1*, *CHST11*, *TMEM263*
Albumen height	7	24.1 … 24.2	WC	24818, 24820, 24821, 24953	
	15	6.2	RW/WC	24953	
Antibody response to SRBC antigen ^3^	2	69.8 … 72.6	RW, WC	14397	
	5	29.6 … 32.9	WC	14402	
Average daily gain	4	70.8 … 72.0	WC	15318	
Body temperature	2	123.0 … 123.7	RW	30853	
Body weight, 28 days	7	6.7 … 8.0	WC	160884	
	26	3.8	RW/WC	95418	
Body weight, 35–49 days	3	29.2 … 29.8	RW, WC	24377, 24378, 24379, 30854	
Body weight, 56 days	5	29.6 … 32.9	WC	153752	
		31.1 … 32.8	RW	153753	
		31.3 … 32.3	RW/WC	153754	
Breast muscle percentage	1	41.1 … 44.5	RW	95427	
	18	0.02 … 0.8	WC	166767, 166768, 166766	
Breast muscle pH	1	10.7 … 11.3	RW	157157	
		18.1 … 18.7	WC	157158	
	2	24.8 … 26.0	WC	157164	
		69.6 … 70.8	WC	157165	
	4	88.8 … 89.2	RW	157246	
	5	17.8 … 18.8	RW	157176	
	8	7.1 … 7.8	WC	157180	
	9	17.8	RW/WC	157184	
		18.0 … 18.3	RW	157185	
	26	3.8	RW/WC	157206	
Carcass fat content	1	53.4 … 55.6	WC	193637	
	28	4.2 … 5.0	WC	193647, 193655	
Egg production rate	3	12.8 … 13.5	WC	214374	
	13	15.7 … 16.0	RW	172762, 172763, 172764, 172765	
Feather pigmentation	1	53.4 … 55.6	WC	137117, 137118	*NUAK1*
Feed conversion ratio	1	53.4 … 55.6	WC	139668	
	1	75.5 … 76.4	WC	139747	
	3	12.8 … 13.5	WC	139401	
	3	29.2 … 29.8	RW, WC	139333	
	5	17.8 … 18.8	RW	139665, 139577	
	6	9.7	RW/WC	139402, 139404, 139406, 139432–139434, 139504, 139531, 139537, 139543, 139589, 139661, 139709, 139712, 139733, 139743, 139760, 139761, 139781, 139786	
	7	6.7 … 8.0	WC	139435, 139472, 139597, 139598, 139741	
	8	7.1 … 7.8	WC	139410	
	13	15.7 … 16.0	RW	64562	
Feed intake	4	38.0 … 38.6	RW	195036, 195037, 195038, 195039, 195040, 195041, 195042, 195043, 195044, 195045, 195046, 195047, 195048, 195049, 195085, 195087, 195096	
	4	57.7 … 58.4	WC	194985	*BMPR1B*
Shank circumference	8	7.1 … 7.8	WC	213550	
Yolk weight	1	41.1 … 44.5	RW	24938, 24939, 24940	

^1^ Genomic region (in Mb) or SNP (in base pair), identified in present study. ^2^ As shown in QTLdb [68]. ^3^ SRBC antigen, sheep red blood cell antigen.

**Table 7:** Functional annotation and enrichment of gene ontology (GO) terms among the identified genes within the sweep regions as ascertained by DAVID.

Category	GO Term	Count	*p*-Value	FE ^1^	FDR ^2^	Genes ^3^
Annotation cluster 1: Enrichment Score: 2.15						
INTERPRO	IPR001202:WW domain	6	0.003	5.98	1.000	*GAS7*, *HECW2*, *MAGI2*, *TCERG1*, ***WWP1***, *WWTR1*
SMART	SM00456:WW	6	0.003	5.85	0.477	
UP_SEQ_FEATURE	domain:WW 1	4	0.017	7.20	1.000	
UP_SEQ_FEATURE	domain:WW 2	4	0.017	7.20	1.000	
Annotation cluster 2: Enrichment Score: 1.91						
GOTERM_BP_DIRECT	GO:0000187~activation of MAPK activity	8	0.002	4.64	1.000	*CPNE3*, *CXCR4*, *DUSP6*, *HGF*, ***IGF1***, ***INSR***, *NTF3*, ***PIK3CB***, *SEMA3C*, ***SEMA5A***, *THBS1*
GOTERM_BP_DIRECT	GO:0030335~positive regulation of cell migration	8	0.022	2.83	1.000	
GOTERM_BP_DIRECT	GO:0032148~activation of protein kinase B activity	3	0.055	7.77	1.000	
Annotation cluster 3: Enrichment Score: 1.57						
UP_KEYWORDS	Chromophore	3	0.016	14.80	0.971	*CLRN1*, ***CRY1***, *OPN5*, ***RPE65***, *RRH*, ***TGFBI***
UP_KEYWORDS	Photoreceptor protein	3	0.016	14.80	0.971	
GOTERM_BP_DIRECT	GO:0018298~protein-chromophore linkage	3	0.017	14.42	1.000	
UP_KEYWORDS	Sensory transduction	6	0.126	2.25	1.000	
Annotation cluster 4: Enrichment Score: 1.32						
GOTERM_BP_DIRECT	GO:0000187~activation of MAPK activity	8	0.002	4.64	1.000	*CCND1*, ***COL6A2***, *CXCR4*, *DUSP6*, *HGF*, ***IGF1***, ***INSR***, *NTF3*, ***PIK3CB***, *THBS1*
GOTERM_BP_DIRECT	GO:0031093~platelet alpha granule lumen	3	0.266	2.98	1.000	
KEGG_PATHWAY	hsa04510:Focal adhesion	7	0.268	1.59	1.000	

^1^ FE, fold enrichment. ^2^ FDR, false discovery rate. ^3^ Candidate genes found in other studies are shown in bold (see Table S5 for details).

## Data Availability

The genotyping data presented in this study can be shared with the third parties upon approval with the GWMAS Consortium.

## Supplementary Materials

The following are available online at https://www.mdpi.com/article/10.3390/biology10090876/s1. Table S1: Number and overall length of ROHs by ROH length class. Table S2: Top 0.1% SNPs by *F*_ST_ values. Table S3: ROH islands identified in genome of the White Cornish and (WC) and Russian White (RW) chicken populations. Table S4: Regions in genome of the White Cornish (WC) and Russian White (RW) chicken breeds as identified by the hapFLK analysis. Table S5: Summary of selective sweeps and candidate SNPs observed in the present study as compared with other related investigations. Table S6: Summary of chicken genes and their human orthologs within genomic regions of selective sweeps as retrieved from BioMart (Ensembl Genes release 103). Table S7: Genomic regions identified in the RW and WC chicken breeds, which are overlapped with known associations and QTLs. Figure S1: A plot showing cross-validation (CV) error (*Y*-axis) for different K-values (*X*-axis). Figure S2: Distribution of ROHs within chromosomes. Figure S3: Plots of the chromosome areas containing the hapFLK regions.
