# Supplementary material for: Unveiling Comparative Genomic Trajectories of Selection and Key Candidate Genes in Egg-Type Russian White and Meat-Type White Cornish Chickens
Source: Biology (Basel). 2021 Sep 6;10(9):876. doi: 10.3390/biology10090876 (PMC8469556; doi:10.3390/biology10090876)
Supplement: Supplementary file 1 [file biology-10-00876-s001.zip › Fig_S2_ROH.pdf]

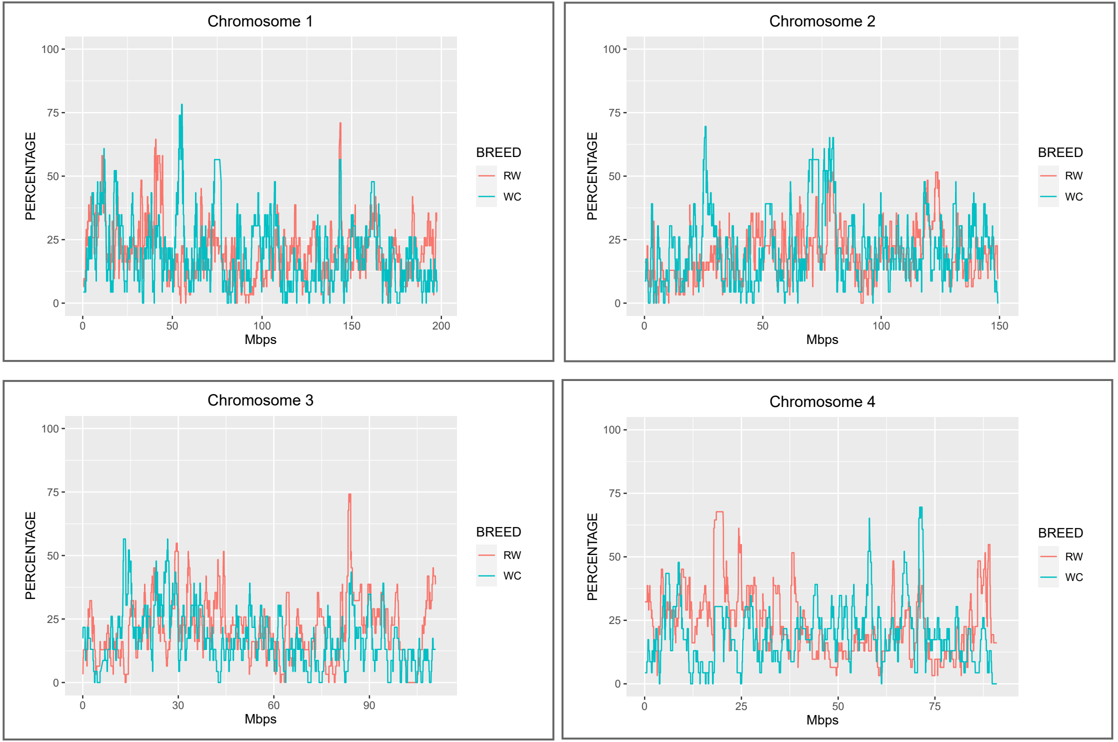
Chromosome 1
Chromosome 2
Chromosome 3
Chromosome 4
PERCENTAGE
Mbps
BREED
RW
WC
100
75
50
25
0
0
50
100
150
200
30
60
90

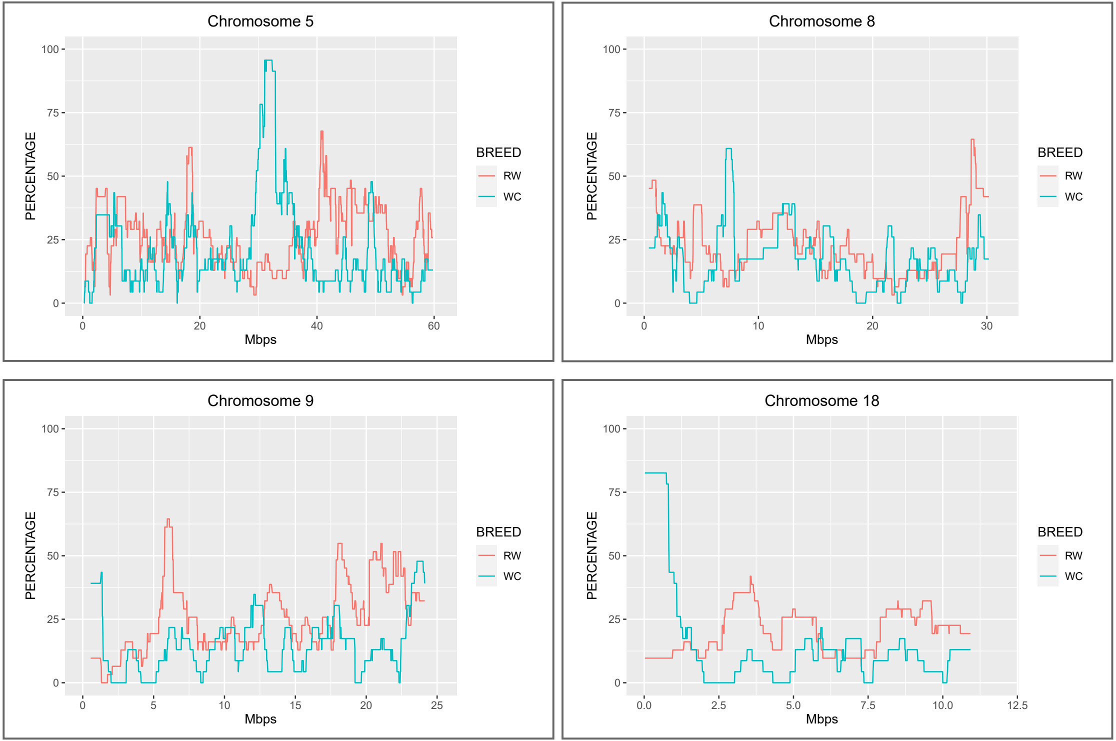
Chromosome 5
Chromosome 8
Chromosome 9
Chromosome 18
PERCENTAGE
Mbps
BREED
RW
WC
100
75
50
25
0
20
40
60
10
30
5
15
0.0
2.5
5.0
7.5
10.0
12.5
