# Supplementary figures and images for: Unveiling Comparative Genomic Trajectories of Selection and Key Candidate Genes in Egg-Type Russian White and Meat-Type White Cornish Chickens
Source: Biology (Basel). 2021 Sep 6;10(9):876. doi: 10.3390/biology10090876 (PMC8469556; doi:10.3390/biology10090876)

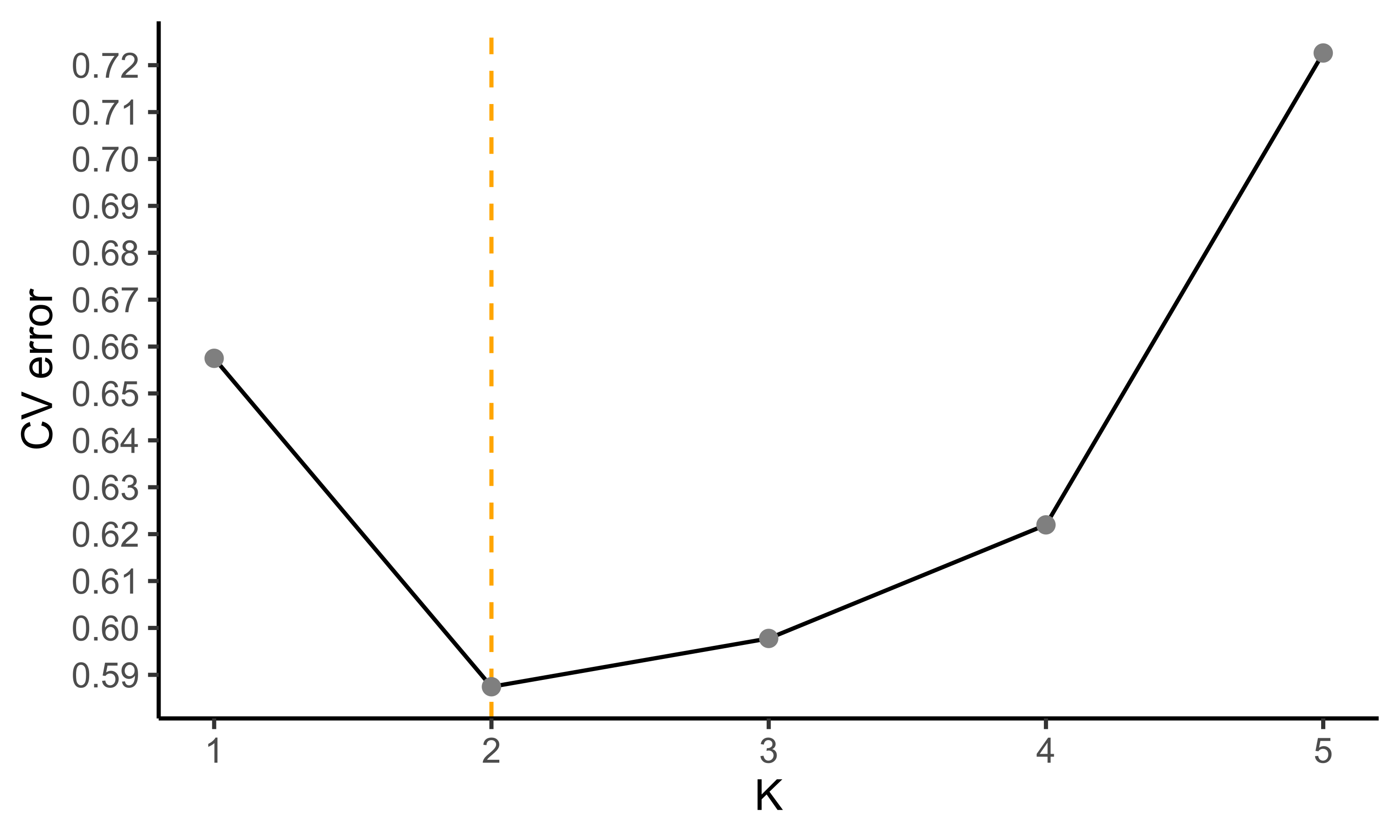

Supplement: Supplementary file 1 [file biology-10-00876-s001.zip › Fig_S1_CV_error.png]

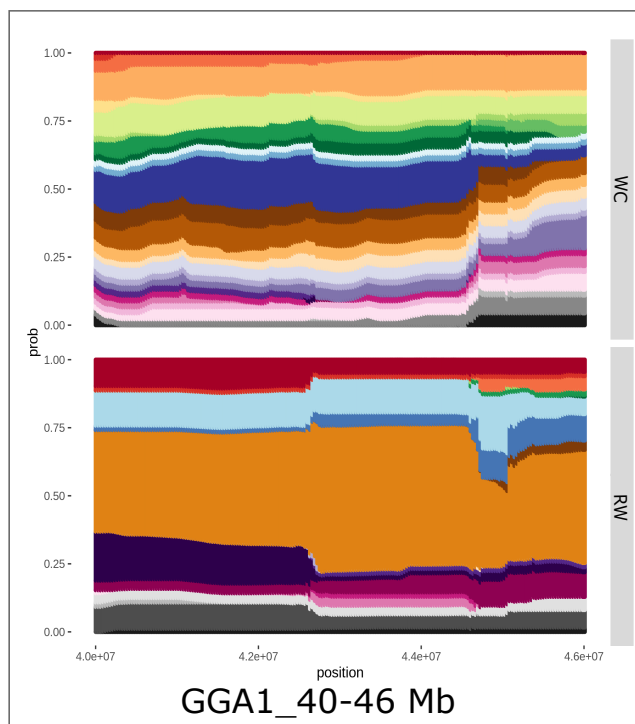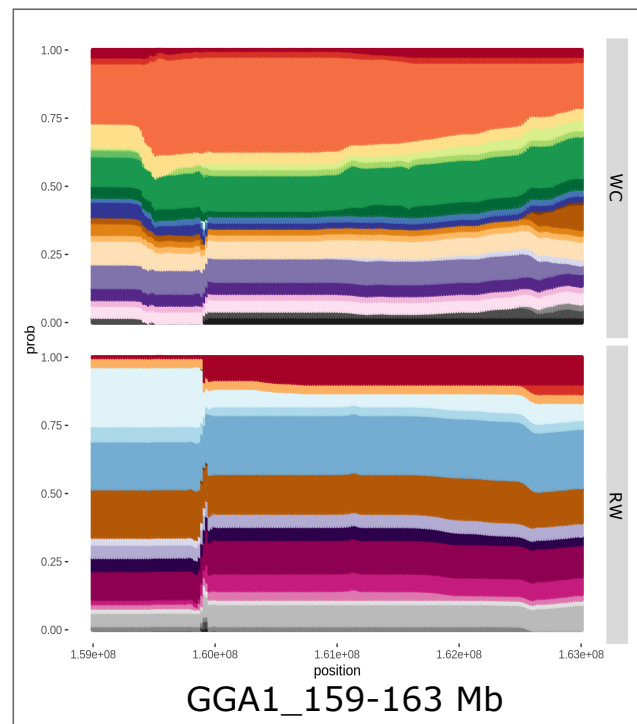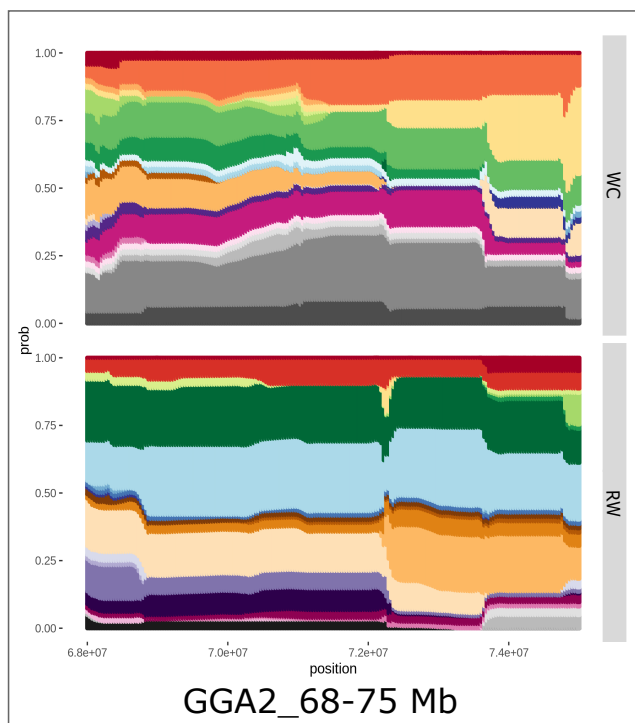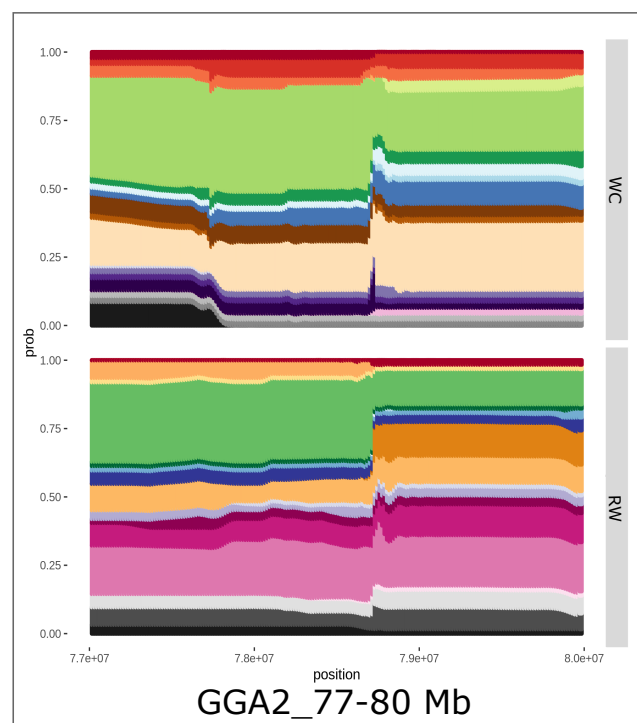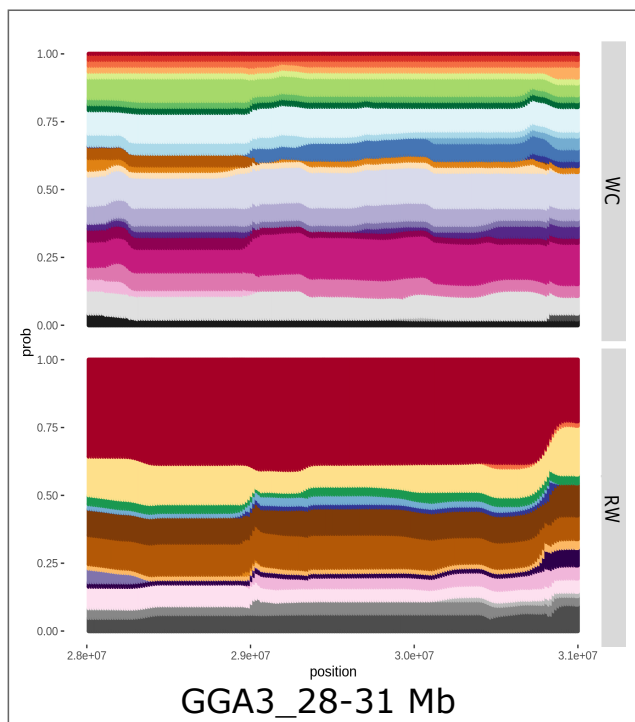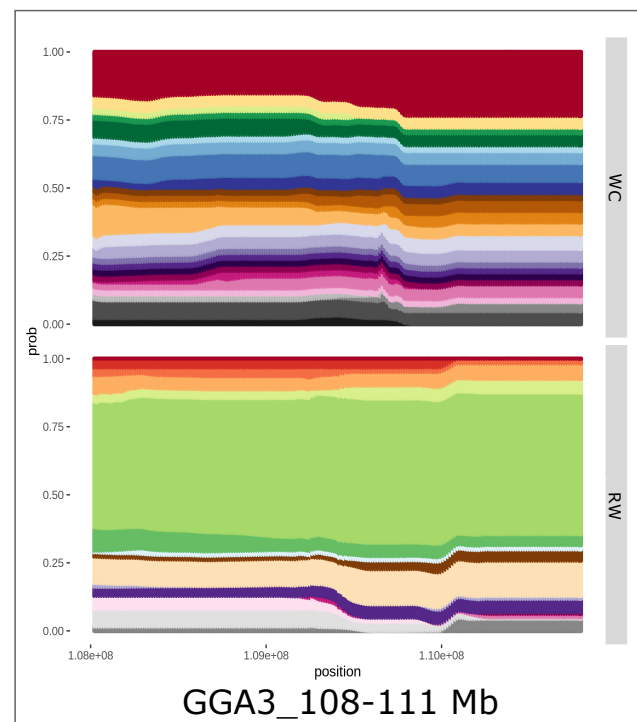

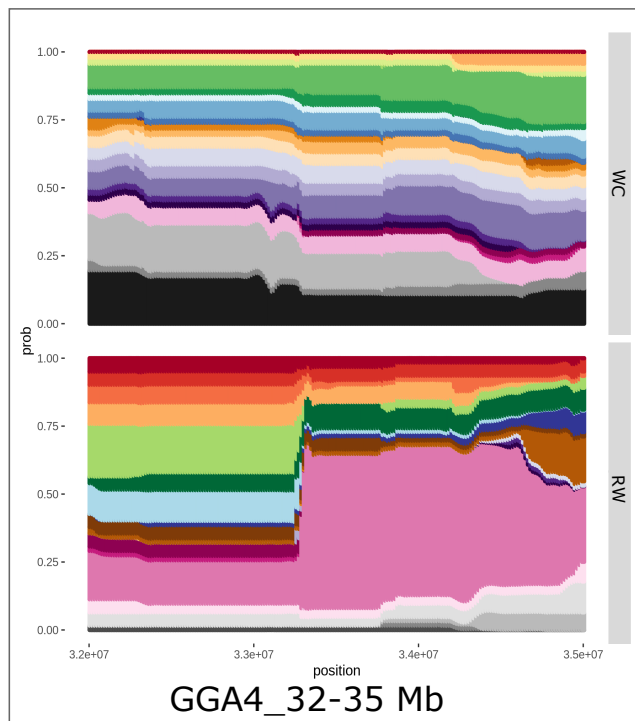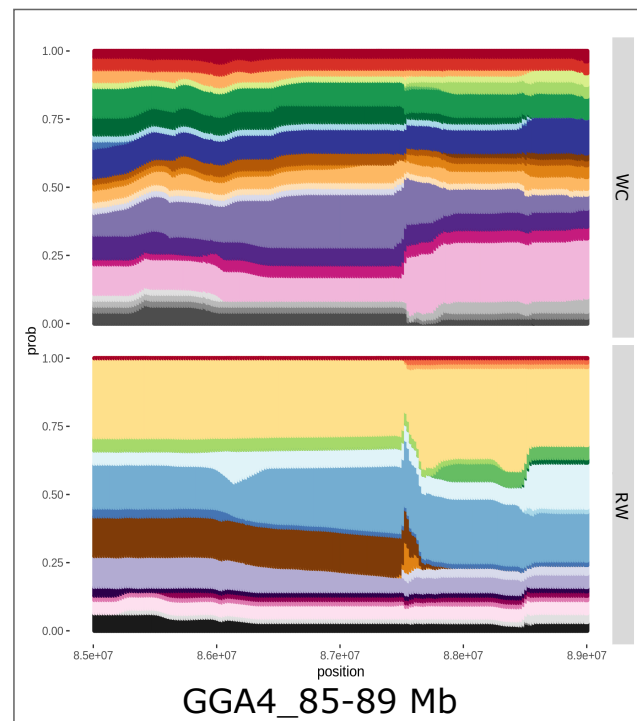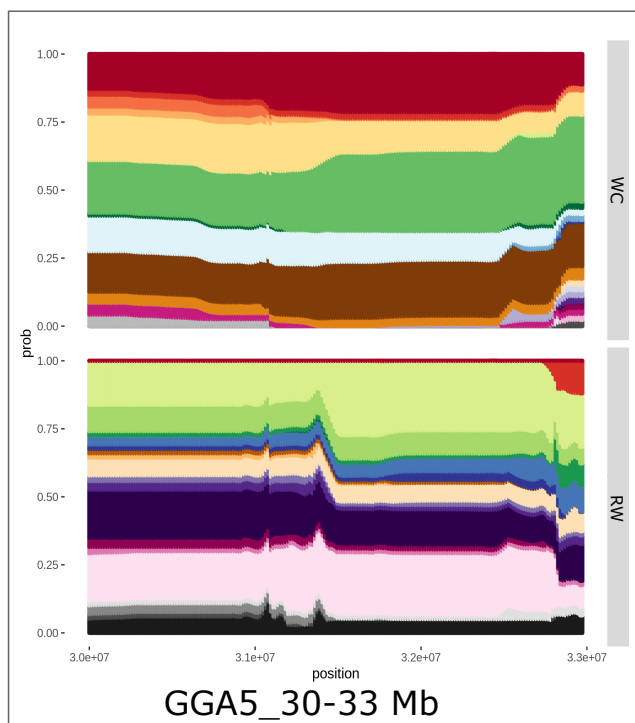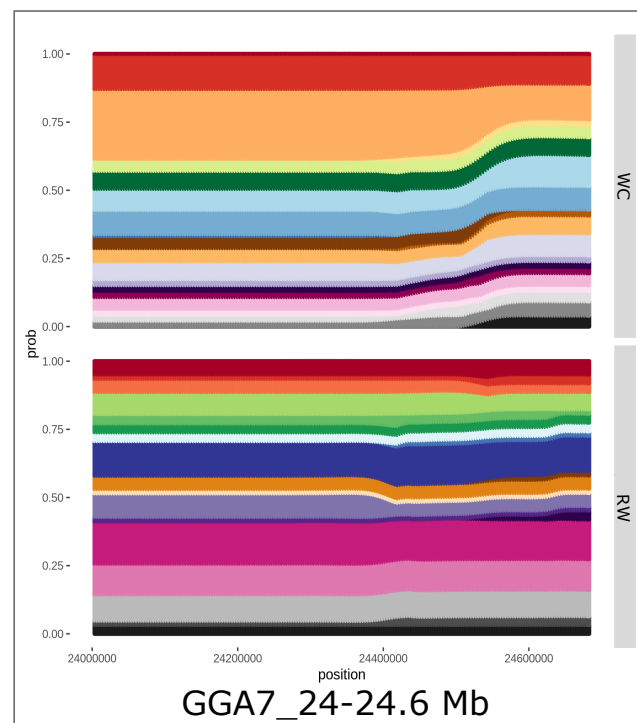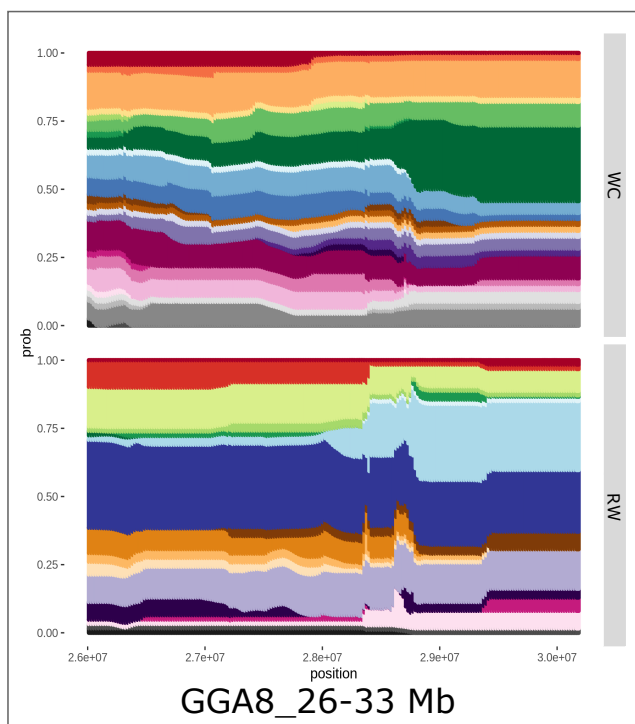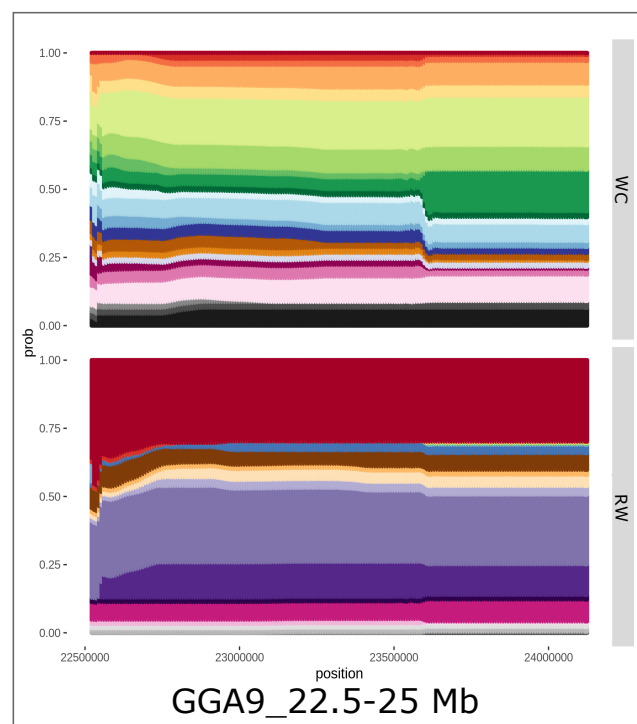

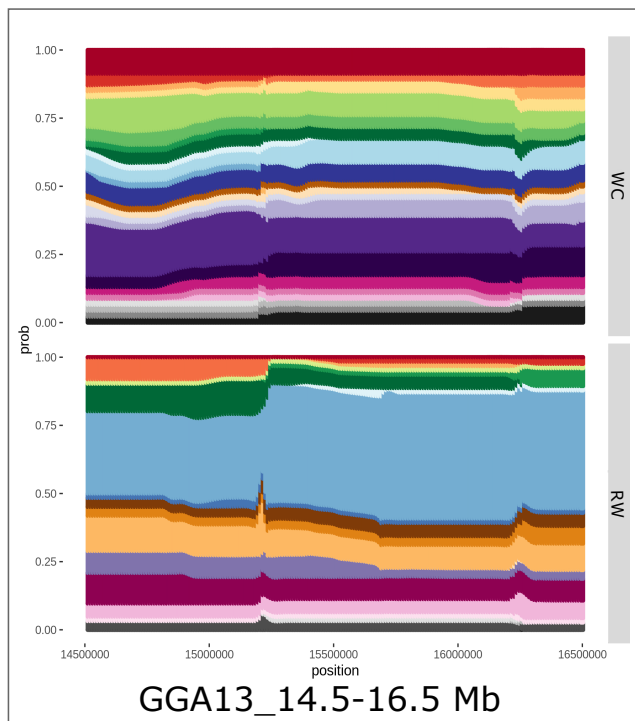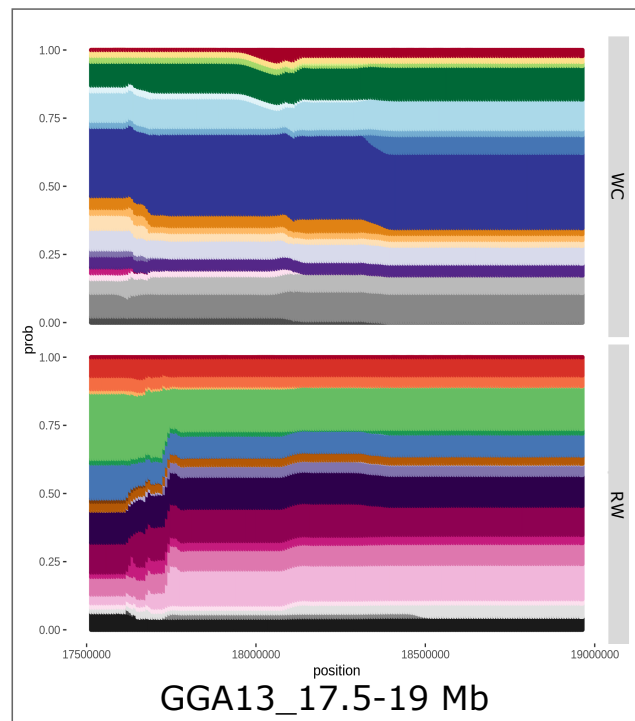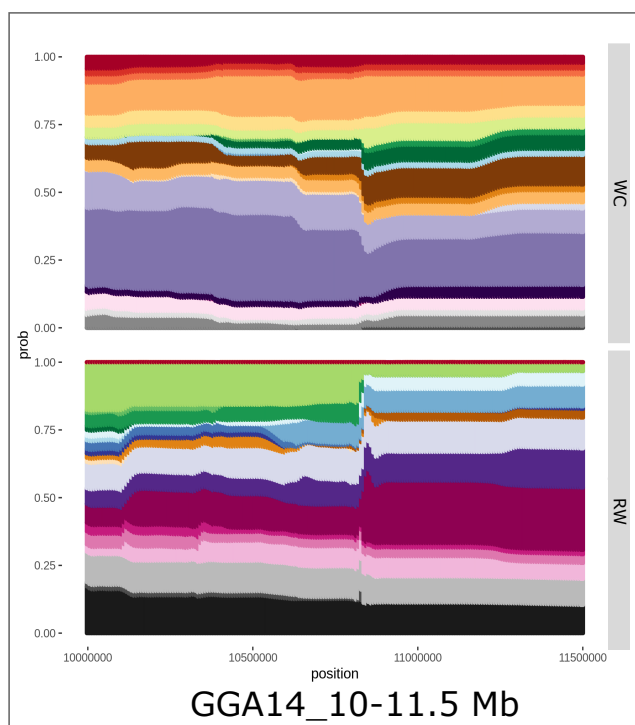

Supplement: Supplementary file 1 [file biology-10-00876-s001.zip › Fig_S3_hapFLK.pdf]
